# CD71 + erythroid cells promote intestinal symbiotic microbial communities in pregnancy and neonatal period

**DOI:** 10.1186/s40168-024-01859-0

**Published:** 2024-07-30

**Authors:** Petya Koleva, Jia He, Garett Dunsmore, Najmeh Bozorgmehr, Julia Lu, Maia Huynh, Stephanie Tollenaar, Vivian Huang, Jens Walter, Sing Sing Way, Shokrollah Elahi

**Affiliations:** 1 School of Dentistry, Division of Foundational Sciences, Faculty of Medicine and Dentistry, Edmonton, Canada; 2 Department of Agricultural, Food & Nutritional Sciences, Edmonton, University of Alberta, Edmonton, Canada; 3 Division of Gastroenterology, Faculty of Medicine and Dentistry, University of Alberta, Edmonton, Canada; 4 Division of Gastroenterology, Mount Sinai Hospital, Toronto, Canada; 5 School of Microbiology and Department of Medicine, APC Microbiome Ireland, University College Cork, National University of Ireland, Cork, Ireland; 6 Centre for Inflammation and Tolerance, Cincinnati Childrens Hospital, College of Medicine, University of Cincinnati, Cincinnati, OH USA; 7 Li Ka Shing Institute of Virology, University of Alberta, Edmonton, Canada; 8 Glycomics Institute of Alberta, University of Alberta, Edmonton, Canada; 9 Women and Children’s Health Research Institute, University of Alberta, Edmonton, Canada; 10 Alberta Transplant Institute, Edmonton, AB Canada; 11 7020G Katz Group Centre for Pharmacology and Health Research, 11361-87Th Ave NW, Edmonton, AB T6G2E1 Canada

## Abstract

**Background:**

The establishment of microbial communities in neonatal mammals plays a pivotal role in shaping their immune responses to infections and other immune-related conditions. This process is influenced by a combination of endogenous and exogenous factors. Previously, we reported that depletion of CD71 + erythroid cells (CECs) results in an inflammatory response to microbial communities in newborn mice.

**Results:**

Here, we systemically tested this hypothesis and observed that the small intestinal lamina propria of neonatal mice had the highest frequency of CECs during the early days of life. This high abundance of CECs was attributed to erythropoiesis niches within the small intestinal tissues. Notably, the removal of CECs from the intestinal tissues by the anti-CD71 antibody disrupted immune homeostasis. This disruption was evident by alteration in the expression of antimicrobial peptides (AMPs), toll-like receptors (TLRs), inflammatory cytokines/chemokines, and resulting in microbial dysbiosis. Intriguingly, these alterations in microbial communities persisted when tested 5 weeks post-treatment, with a more notable effect observed in female mice. This illustrates a sex-dependent association between CECs and neonatal microbiome modulation. Moreover, we extended our studies on pregnant mice, observing that modulating CECs substantially alters the frequency and diversity of their microbial communities. Finally, we found a significantly lower proportion of CECs in the cord blood of pre-term human newborns, suggesting a potential role in dysregulated immune responses to microbial communities in the gut.

**Conclusions:**

Our findings provide novel insights into pivotal role of CECs in immune homeostasis and swift adaptation of microbial communities in newborns. Despite the complexity of the cellular biology of the gut, our findings shed light on the previously unappreciated role of CECs in the dialogue between the microbiota and immune system. These findings have significant implications for human health.

Video Abstract

**Supplementary Information:**

The online version contains supplementary material available at 10.1186/s40168-024-01859-0.

## Introduction

Mounting preclinical and clinical evidence strongly supports the crucial role of the gut microbiota in the development of a healthy immune system, particularly in the early stage of life [1–4]. The perturbed interaction of this coordinated and melodious relationship, often referred to as dysbiosis, is associated with various diseases such as inflammatory bowel disease (IBD) [5, 6], type 1 diabetes [7], asthma, [8] and necrotizing enterocolitis in infants [9]. Although the crucial role of the microbiome in the neonatal period is not in question, the mechanism by which the host tolerates these new allies is still poorly understood.

As the fetus remains sterile in healthy pregnancies [10, 11], the newborn is exposed to a massive influx and colonization of mucosal surfaces or skin with microbial communities at birth and postnatally. The microbial sensing at the mucosal surfaces is required and tightly regulated to safeguard a mutualistic relationship between the host and its microbial communities [3]. Otherwise, a dysregulated innate immune recognition of commensal microorganisms will result in an inflammatory response with potential spontaneous inflammatory response, as validated by the loss of host-microbiota homeostasis in the absence of microbial recognition by Toll-like receptors (TLRs) [12–14]. Thus, intestinal epithelial cells integrate microbiota-induced signaling into a balance at the host-microbial interface, which is composed of mucosa, host-defense peptides (HDPs), and harmonization of cellular response [3].

Antimicrobial peptides (AMPs) or HDPs are broadly expressed by different immune and non-immune cells [15, 16]. Defensins and cathelicidins are the predominant AMPs, playing vital roles in governing the microbial communities and maintaining homeostasis at the mucosal surfaces of the gut [17]. For example, severe inflammation and mucosal disruption are reported in the absence of mCRAMP (a mouse cathelicidin) [18]. Similarly, lack of cryptdins (α-defensin) enhances susceptibility to bacterial infections [19] and compromises intestinal barrier integrity in animal models [20].

Although the role of microbial communities in the development of different innate immune components is well-defined, [21] it’s unclear how the innate immune system is regulated upon the acquisition of the newborn’s first colonizers after birth.

Recently, we reported the abundance of erythroid precursors/progenitors defined as CD71^+^ erythroid cells (CECs) in the spleen of neonatal mice and the peripheral blood of human newborns [22–24]. CECs co-express CD71 and TER119 in mice but CD71 and CD235a in humans with distinctive immunosuppressive properties [23, 25]. Previously, we showed that CECs-mediated susceptibility to infection was counterbalanced by protection against immune cell activation in the neonatal intestine following colonization with commensal microbial communities [23].

Similarly, we found an expansion of CECs in the peripheral blood and spleen of humans and mice during pregnancy, respectively [26, 27], however, this was not the case in the peripheral blood of pregnant women with IBD. Hence, this factor may contribute to microbiome alterations and pro-inflammatory milieu in the gut [27] of IBD patients as reported in a mouse model of colitis [28]. Even though such studies suggest a potential role for CECs in establishing microbe-immune homeostasis in the gut, this hypothesis has never been systemically tested.

Therefore, we investigated the frequency of CECs in different gut compartments in neonatal and adult mice. Also, we characterized and compared the biological properties of the gut versus spleen CECs. By depleting CECs, we determined immunological changes in intestinal tissues of neonatal and adult mice. Finally, by using bacterial qPCR and 16S rRNA sequencing, we profiled the impact of CECs on the biodiversity and development of microbial communities in the neonatal period and pregnancy in mice.

## Results

### The intestinal *lamina* propria is enriched with CECs during the early stage of life

**Fig. 1:**
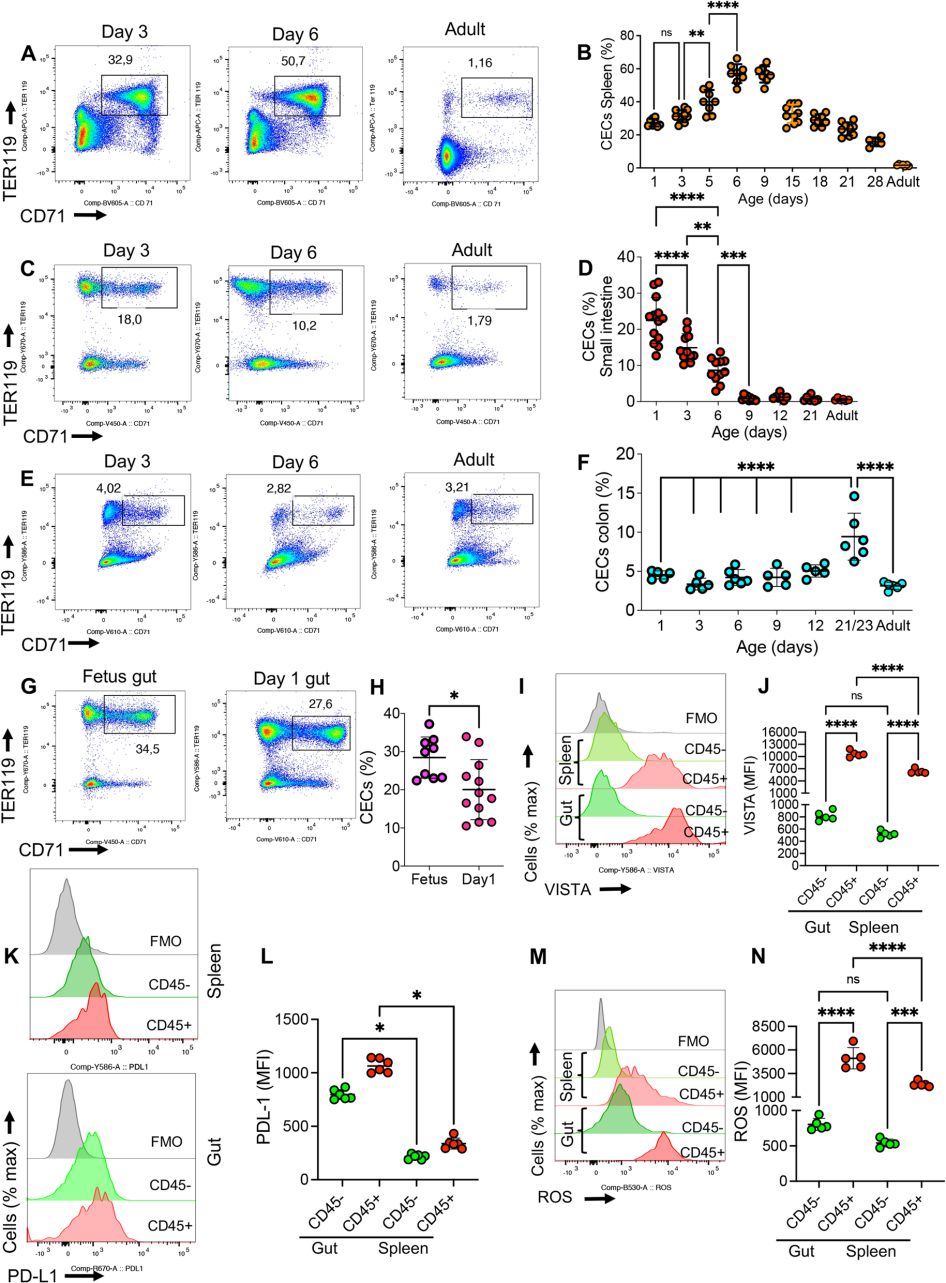
Characterization of CECs in the gut lamina propria. **A** Representative plots, and **B** cumulative data of % CECs in the spleen of mice over time. **C** Representative plots, and **D** cumulative data of % CECs in lamina propria of the small intestine in newborn and adult mice. **E** Representative plots, and **F** cumulative data of % CECs in colonic lamina propria in newborn and adult mice. **G** Representative plots, and **H** cumulative data of % CECs in lamina propria of the small intestine of fetus and one-day-old mice. **I** Histogram plots, and **J** cumulative data of the mean fluorescence intensity (MFI) for VISTA in CD45^+^/CD45^−^CECs from the small intestinal (gut) and spleen samples collected from one-day-old mice. **K** Histogram plots, and **L** cumulative data of the intensity of PD-L1 expression in intestinal and spleen CD45^+^/CD45^−^CECs of one-day-old mice. **M** Histogram plots, and **N**) cumulative data of ROS production by CD45^+^/CD45.^−^CECs from the small intestine (gut) and spleen of one-day-old mice. Each symbol represents an animal. Fluorescence minus one (FMO). Data are collected from multiple independent experiments. Results are presented as standard deviation (SD) and *P* values were calculated using two tailed, Mann–Whitney *t* test (H) or One-way ANOVA test (B,D, F,J,L,N). (*P* < 0.05 (*), *P* ≤ 0.01 (**), *P* ≤ 0.001 (***), and *P* ≤ 0.00001 (****). not significant (ns)

Consistent with our previous reports [23, 25], we found that CECs are highly abundant in the spleen of neonatal mice, with the highest frequency occurring at day 6–9, but declining gradually during the postnatal development (Fig. 1A,B). Our previous observations suggested that CECs protect against excessive inflammation triggered by the colonization with microbial communities in newborns [23]. However, their presence in the gut and possible interaction with the microbiota remained unexplored. Here, for the very first time, we analyzed the presence of CECs in the lamina propria of the small intestine and colon of neonatal mice. We found that the small intestine was enriched with CECs (Fig. 1C,D and the gating strategy, S Fig. 1A). While the frequency of CECs at day 1 was approximately 20% of total isolated lymphocytes in the small intestinal tissue, in the colon it was notably lower (around > 5%) (Fig. 1E,F). The pattern of CECs’ frequency in the small intestine showed an opposite trend compared to the spleen during the early days of life. While the frequency of CECs in the spleen increased with age and reached to its peak by day 6–9 (Fig. 1B), the small intestine had the highest frequency of CECs at day 1 but gradually began to decline by age (Fig. 1D). However, the proportion of CECs in colonic lymphoid tissues remained consistently low over time, except at day 21–23, when we observed a significantly higher frequency of CECs compared to younger and adult mice (Fig. 1F and S Fig. 1A). Considering that this is the period when newborns are weaned, we speculate that the increased percentage of CECs at day 21–23 might be due to changes in solid food intake and possible alterations in the gut microbial composition. Given that CECs had the highest proportion in the small intestine immediately after birth, we hypothesized that they might also be present in the gut during fetal development. In agreement, we observed a high abundance of CECs in the fetal gut (E18.5–19.5) and their proportions were significantly higher compared to 1-day-old newborns (Fig. 1G,H).

To further analyze the phenotypical characteristics of CECs, we measured the expression of PD-L1, VISTA, and reactive oxygen species (ROS) [26, 29] in intestinal CECs of 1-day-old mice in comparison with their counterparts in the spleen. As anticipated [30, 31], CD45^+^CECs regardless of their source (e.g. the spleen or gut) exhibited a greater expression level of VISTA, PD-L1, and ROS compared to their CD45^−^ counterparts (Fig. 1I-N). The most notable observation was that intestinal CD45^+^CECs had significantly higher VISTA, PD-L1, and ROS expression levels than their CD45^+^ counterparts in the spleen of neonatal mice (Fig. 1I-N). Although VISTA and ROS expression levels were comparable in intestinal and splenic CD45^−^CECs, this subpopulation had significantly greater expression levels of PD-L1 in the gut of neonatal mice (Fig. 1I-N). These observations not only demonstrate the physiological abundance of CECs in the neonatal intestine but also indicate clear differences in the biological properties of intestinal and splenic CECs in the newborn.

### Erythropoiesis niches are present in the neonatal intestine

**Fig. 2:**
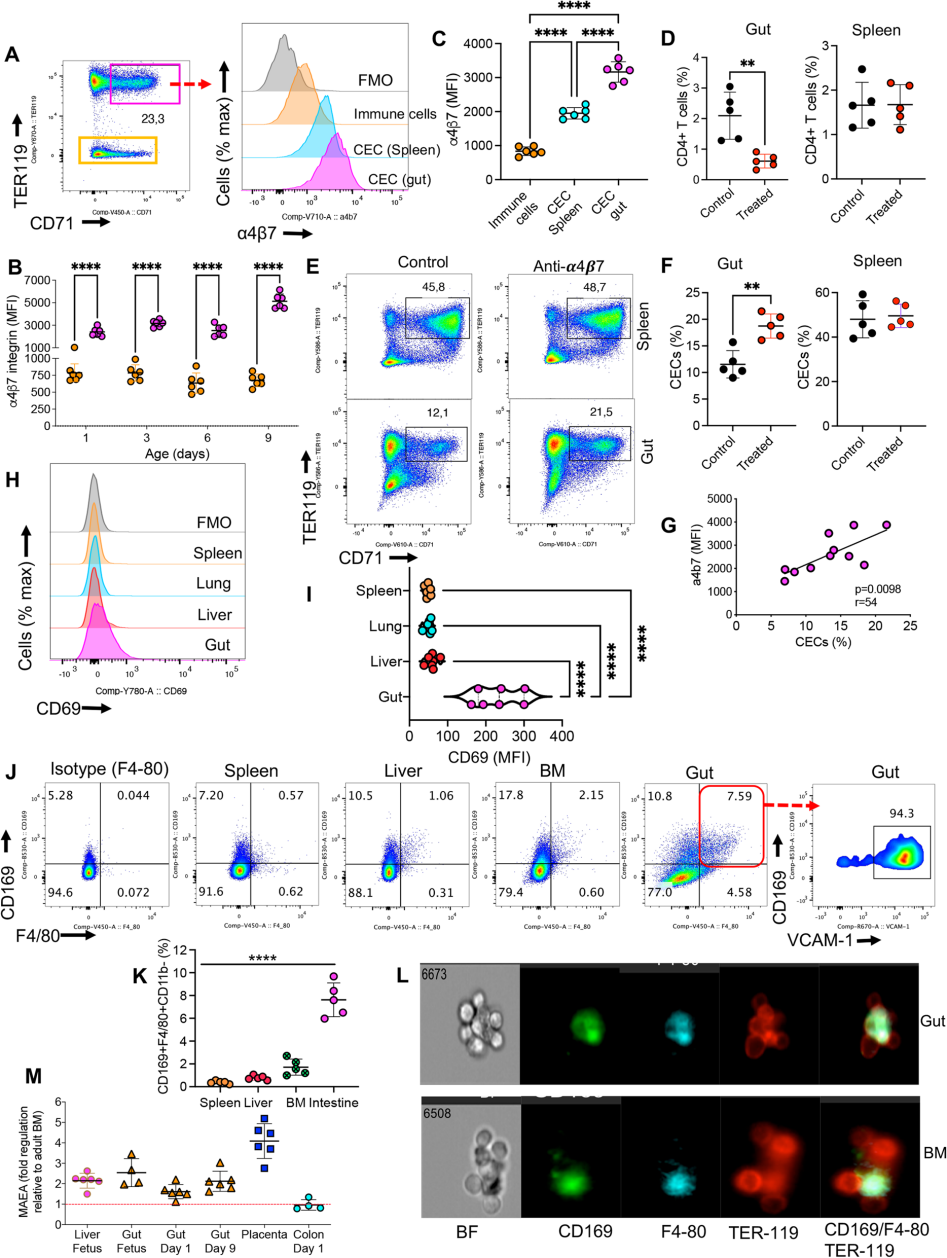
Regulated migration or site-specific hematopoiesis in the gut. **A** Plots showing α4β7 expression in CECs and non-CECs in the spleen and gut of a 3-day-old mouse. **B** Cumulative data of the mean fluorescence intensity (MFI) of α4β7 expression in CECs and non-CECs from the gut of mice over time, and **C** in splenic immune cells versus splenic and intestinal CECs. **D** Cumulative data of the % of CD4^+^ T cells in the small intestine and spleen of 4-day-old mice one day post-treatment with the anti-α4β7 antibody (150 μg/mouse). **E** Representative plots, and **F** cumulative data of the % of CECs in the spleen and intestinal tissues of 4-day-old mice one day post-treatment with the anti- α4β7 antibody. **G** Correlation analysis of % CECs and MFI of α4β7 in the small intestine (gut) of 3-day-old mice. **H** Representative plots, and **I** cumulative data of the MFI for CD69 in CECs from the gut, liver, lungs, and spleen of 3-day-old mice. **J** Representative plots, and **K** cumulative data of central macrophages in the adult bone marrow (BM), neonatal spleen, liver, and small intestine (gut) obtained from 3-day-old mice. **L** Representative Image Stream analysis showing an erythroblastic island in the small intestine of a 3-day-old mouse and the BM of an adult mouse as characterized by a central macrophage (CD11b^−^CD169^+^F4-80^+^) surrounded by CECs/RBCs (TER119 +). **M** Fold regulation of Maea gene (macrophage erythroblast attacher) in the fetal (liver and gut), neonatal (1, 9 days old), and placenta tissues relative to the adult mouse BM. Results are presented as SD and *P* values were calculated using two tailed, Mann–Whitney *t* test (D, F), One-way ANOVA test (B, C, I, K) or the Spearmen correlation analysis (G). (*P* < 0.05 (*), *P* ≤ 0.01 (**), *P* ≤ 0.001 (***), and *P* ≤ 0.00001 (****). Fluorescence minus one (FMO). Data are collected from multiple independent experiments

**Fig. 3:**
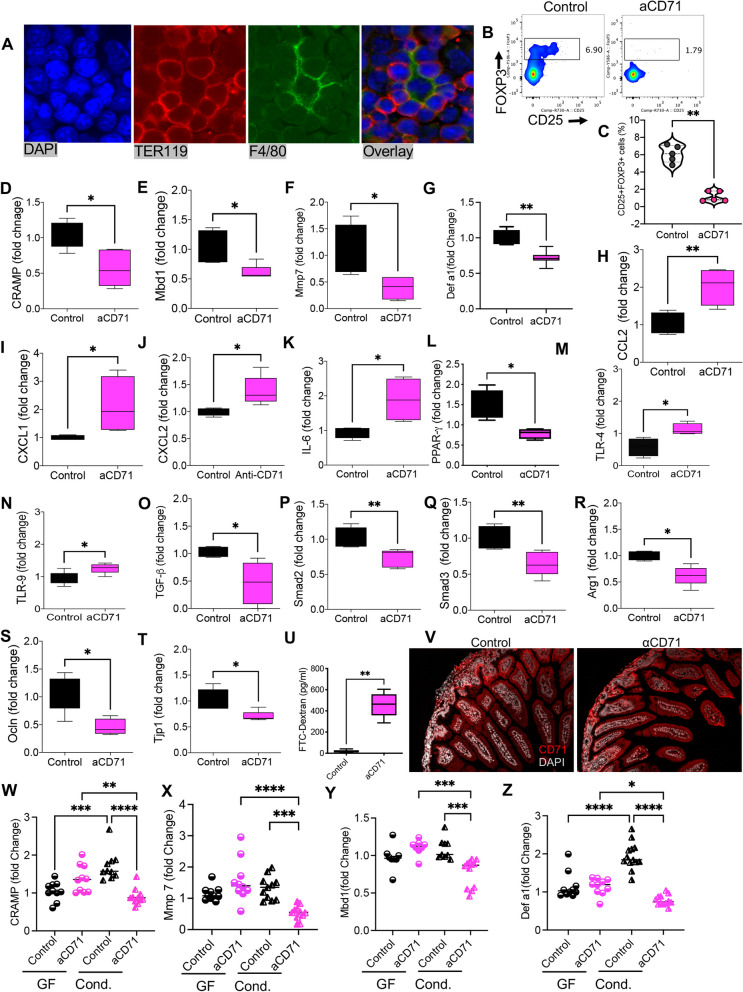
Depletion of CECs results in altered epithelial immune homeostasis and intestinal inflammatory response. **A** Representative immunofluorescence staining for erythropoiesis niches, DAPI (blue), TER119 (red), F4/80 (green).** B** Representative flow cytometry plots, and **C** cumulative data of percentages of Tregs in the small intestine of control and anti-CD71 treated mice. **D** Fold regulation of antimicrobial peptides (AMPs) associated genes (Cramp, cathelicidin), **E** Mbd1 (mouse beta-defensin-1), **F** Mmp7 -matrix metallopeptidase 7, and **G** alpha defensin-1 (Defα1) in treated neonatal mice with the anti-CD71 antibody vs controls. **H** Relative expression level of CCL2, **I** CXCL1, and **J** CXCL2) in small intestinal tissues collected from isotype control or anti-CD71 treated mice. **K** Relative gene expression of IL-6, **L** PPAR-γ, **M** TLR-4, **N** TLR-9, **O** TGF-β, **P** Smad2, **Q** Smad3, **R** arginase I, **S** tight junctions (Ocln, occluding), and **T** Tjp1-tight junction protein in intestinal tissues of mice treated with the anti-CD71 antibody versus controls. **U** Concentration of fluorescein-isothiocyanate-dextran (FITC-dextran) in serum samples collected from the anti-CD71 or isotype control treated 2-day post-treatment with the anti-CD71 antibody vs controls. **V** Immunofluorescence localization of CD71 receptor (red) and DAPI (white) in small intestines of treated with the anti-CD71 and controls. **W** Fold regulation of AMPs-associated genes CRAMP, **X** Mmp7, **Y** Mbd1, and **Z** Defα 1 in germ-free (GF) mice treated with the anti-CD71 antibody or isotype treated (control) in GF condition or treated/control but cohoused in SPF condition (cond) for 24 h before tissue collection. Each symbol denotes data from an individual animal and obtained from multiple independent experiments. Gene expression studies are from at least 6 animals/groups. The fold change of targeted genes was calculated by the 2.^−ΔΔCt^ method. The expression levels of the respective genes in samples from the 4-day control group were used as calibrators, and β-actin was employed as a reference gene. *P* values were calculated using two tailed, Mann–Whitney *t* test (C-U), Kruskal–Wallis analysis with Dunn’s multiple comparisons test (W-Z) (*P* < 0.05 (*), *P* ≤ 0.01 (**), *P* ≤ 0.001 (***), and *P* ≤ 0.00001 (****). Anti-CD71 (aCD71). not significant (ns). Conditioned (Cond)

**Fig. 4:**
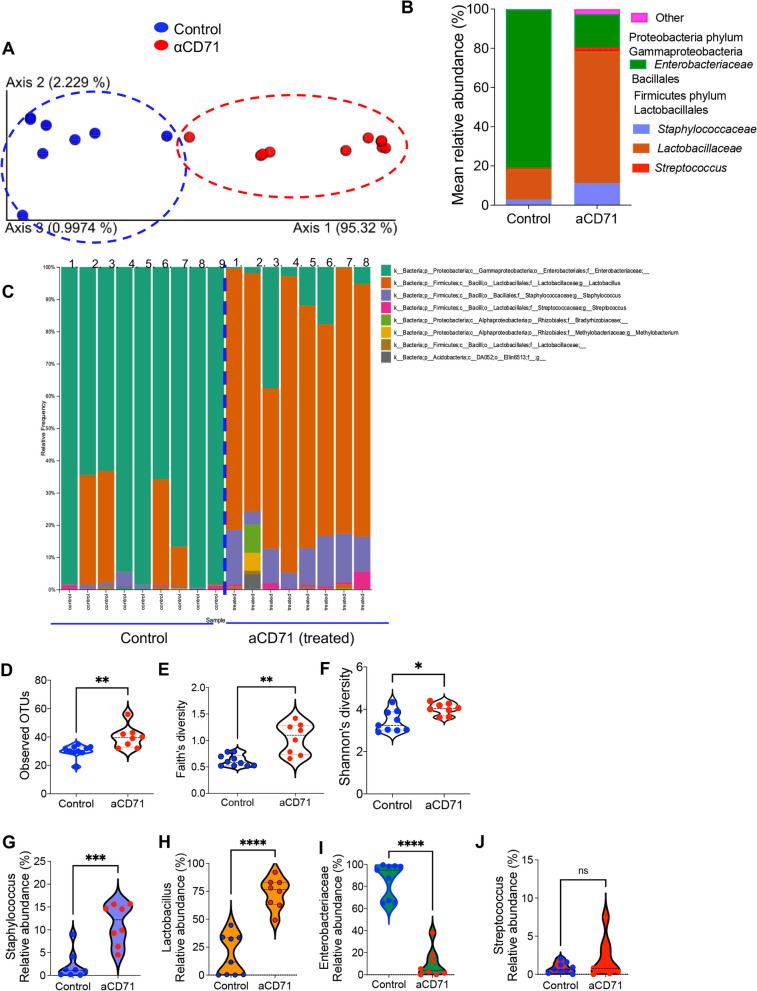
Depletion of CECs resulted in microbial dysbiosis in the gut. **A** Principal Coordinates Analysis (PCoA) of weighted UniFrac matrix (beta diversity measure) identifying differences of microbial communities between control and anti-CD71-treated mice once antibody was administered into 3-day-old pups and samples were collected 1-day post-treatment. Each symbol represents an individual animal. The red color indicates the anti-CD71 treated pup, whereas the blue color depicts the control pup. **B** Cumulative data of comparison of small intestinal microbiota at the family level by treatment in experimental animals. **C** Comparison of small intestinal microbiota at the family level in individual animals (control vs treated with the anti-CD71 antibody). **D** Observed OTUs for anti-CD71-treated versus control mice. **E** Comparison of alpha diversity measures (Faith’s diversity index), and **F** Shannon’s diversity between control and treated mice. treatment and age. **G** The abundance of *Staphylococcaceae* dominant bacteria, **H** *Lactobacillus* bacteria, **I** *Enterobacteriaceae* bacteria, and **J** *Streptococcaceae* in the small intestine of control and anti-CD71 treated mice. Results are presented as SD and *P* values were calculated using two tailed, Mann–Whitney *t* test (**D-J**). (*P* < 0.05 (*), *P* ≤ 0.01 (**). Anti-CD71 (aCD71). not significant (ns)

**Fig. 5:**
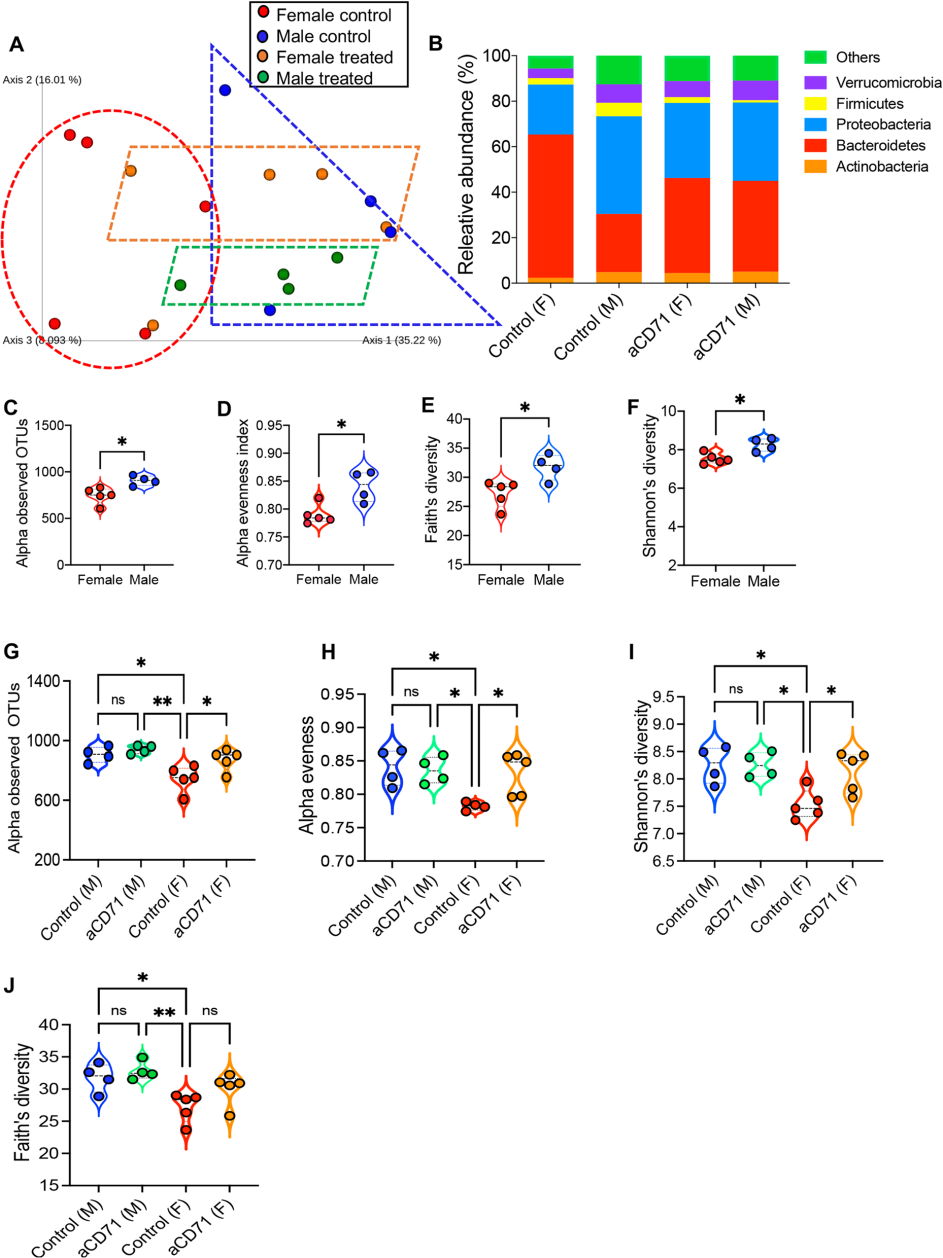
Depletion of CECs at day 3 was associated with prolonged changes in microbial communities. **A** Principal Coordinates Analysis (PCoA) of weighted UniFrac matrix (beta diversity measure) identifying differences of microbial communities between male & female controls, control & anti-CD71-treated mice (males & females) once treated at day 3 and analyzed at day 35 of age. Each symbol represents an individual animal. **B** Cumulative data of comparison of small intestinal microbiota at phylum level by sex and treatment in experimental animals. **C** Observed OTUs for female versus male control mice. **D** Comparison of alpha evenness index between female vs male control mice. **E** Faith’s diversity index, and **F** Shannon’s diversity index between female vs male control mice. **G** Observed OTUs for control vs anti-CD71 treated female/male mice. **H** Comparison of alpha evenness index between control vs anti-CD71 treated female/male mice. **I** Shannon’s diversity index and **J** Faith’s diversity index between control vs treated female/male mice. *P* values were calculated using two tailed, Mann–Whitney *t* test (**C-F**), One-way ANOVA (G-J). (*P* < 0.05 (*), *P* ≤ 0.01 (**), not significant (ns)

Previously, we suggested that splenic CECs may migrate to the gut to inhibit the rapid innate immune response to microbial communities in the newborn [30]. However, as described in Fig. 1, CECs in the gut appear to be different in terms of their biological properties than their counterparts in the spleen. To better understand the source of intestinal lamina propria CECs during the early postnatal period, we assessed their gut-homing receptors. Hence, we measured the surface expression of α4β7 integrin, the lymphocyte trafficking molecule, in CECs. These analyses revealed that intestinal CECs consistently and significantly had higher α4β7 expression compared to other immune cell lineages (non-CECs) at different ages (Fig. 2A,B). Of note, the intensity of α4β7 in CECs from the gut was significantly greater than those in the spleen (Fig. 2A,C). Considering that the recruitment and maintenance of immune cells into the gut mucosal tissues are mediated by the interaction of cellular α4β7-integrin and MAdCAM-1 [32]. We hypothesized this might be the case for CECs and decided to modulate their trafficking into the gut by treating 3-day-old mice with the anti-α4β7 neutralizing antibody. As expected, the anti-α4β7 antibody treatment substantially decreased the presence of CD4^+^ T cells in the intestinal tissues without impacting the frequency of their siblings in the spleen (Fig. 2D). Surprisingly, this treatment significantly increased the percentages of CECs in intestinal tissues but not in the spleen (Fig. 2E,F). Moreover, we assessed the effects of depleting CECs, using the anti-CD71 antibody (S Fig. 1B), [23, 25] on the mRNA expression of α4β7 in intestinal tissues of treated mice. These studies revealed a significant downregulation in α4β7 mRNA in the intestinal tissues but an upregulation of its ligand, MAdCAM1, possibly as a compensatory mechanism (S Fig. 1C,D). Our observations suggest that the anti-α4β7 antibody prevented the migration of CD4^+^ T cells into the intestinal tissues but not CECs. Moreover, we found a positive correlation between the percentages of CECs and the intensity of α4β7 expression in intestinal CECs of neonatal mice (day 3) (Fig. 2G) but this was not the case for the splenic CECs (S Fig. 1E). Also, we assessed the expression of CD69, an early T cell activation marker [33], as the regulator of intestinal inflammation and the gut homing receptor [34] on CECs. We found a higher expression of CD69 in intestinal CECs compared to their counterparts in the spleen, liver, and lungs in day 3 mice (Fig. 2H,I). These observations raised the hypothesis that CECs may not get recruited but are generated in the gut as reported in human intestinal allograft patients [35]. To test this hypothesis, we studied the presence of erythroblastic islands (EBIs) [36] in the neonatal gut, spleen, and liver compared to the bone marrow of adult mice. These islands are niches where central macrophages interact closely with RBCs in their different stages of proliferation/maturation and engulf free nuclei as they are extruded from the reticulocytes [37]. Therefore, we examined the presence of central macrophages (CD11b^−^CD169^+^F4/80^+^VCAM1^+^) according to a recent study [38]. We observed the presence of central macrophages in the intestinal tissues of 3-day-old mice, which was significantly higher compared to other examined niches (Fig. 2J,K and S Fig. 1F). The Image Stream analysis confirmed our observations that CD11b^−^CD169^+^F4/80^+^ cells were surrounded by erythrocytes (TER119^+^ cells) in the small intestine of neonatal and the BM of adult mice (Fig. 2L). The occurrence of erythropoiesis in the gut was further assessed using another indirect approach by measuring the expression level of macrophage erythroblast attacher (Maea). The Maea is expressed by central macrophages but not by erythroblasts (EBs). It is suggested to promote BM erythropoiesis [39, 40] by regulating the maintenance of central macrophages and their interaction with EBs [40]. Therefore, the expression level of the Maea gene was examined in the placental, fetal liver/small intestine compared to the neonatal small intestine/colon and placenta tissues. Interestingly, the Maea mRNA was detectable in the fetal liver and both the fetal and neonatal intestinal tissues (Fig. 2M). The detection of Maea mRNA in the small intestinal tissue provided another indication for the presence of an erythropoiesis niche in the small intestine but not colon. Furthermore, immunofluorescence staining reconfirmed the presence of EBIs, where a central macrophage is surrounded by CECs (Fig. 3A). These EBIs were mainly present in submucosal tissues and sporadically within the villus of neonatal small intestine (S. Figure 2 and S. Figure 3). Of note, in the venules of the intestinal tissues mainly mature RBCs are observed but not CECs (S. Figure 4A). This implies that the gut-associated CECs are mainly generated in the intestinal EBIs and the blood circulation may not be the main source of CECs. As expected, the neonatal spleen was enriched with CECs and EBIs (S. Figure 4B). Taken together, these observations support the concept of erythropoiesis in the gut of conventional neonatal mice. However, germ-free mice despite having a higher proportion of CD169 + cells in their intestinal tissues only a small portion of these cells co-expressed F4-80 but lacked VACM-1 expression (S Fig. 5A). Furthermore, we found that CECs in intestinal tissues of germ-free mice (day 1) had significantly lower expression levels of α4β7 compared to their counterparts in conventional mice (S Fig. 5B). Subsequently, the proportion of CECs in the intestinal tissues of germ-free mice was significantly lower than conventional mice (S Fig. 5C,D) without any difference in the proportion of splenic CECs, as we have reported elsewhere [23].

### CECs prevent intestinal inflammation following microbial colonization

To determine the immunoregulatory roles of CECs in the gut, 3-day-old neonatal mice were subjected to the anti-CD71 antibody administration. When examined 24 h later, we found a significant reduction in the frequency of CECs in the spleen, small intestine, and colon of mice (S Fig. 1B). We noted that depletion of CECs was associated with a hyper-immune activation in the gut evidenced by increased TNF-α expression in CD11b + and CD11c + cells (S Fig. 5E,F). However, this was not the case in the gut of germ-free mice (S Fig. 5G). Of note, we found a significant reduction in the frequency of regulatory T cells (Tregs) in CEC-depleted mice (Fig. 3B,C). This implies that CECs may promote Tregs development as reported elsewhere [29]. These observations suggest that CECs may prevent immune activation following the introduction of the neonatal gut to early microbial colonizers.

Moreover, we investigated possible changes in the mRNA expression level of different factors associated with immune homeostasis in the gut. Since AMPs play an important role in regulating the gut microbial communities while ensuring immune homeostasis [17], we decided to determine whether depletion of CECs influences the expression of AMPs in intestinal tissues. We found that depletion of CECs at day 3 resulted in a significant reduction in mRNA expression for the mouse cathelicidin (CRAMP), mouse beta defensin-1 (Mbd1), MMP7, and α-defensin-1 (Defα1) (Fig. 3D-G). MMP7 is involved in the processing and activation of α-defensins in Paneth cells [41] and based on these observations CECs may modulate AMPs production in Paneth cells. In contrast, Defα5 appeared to be developmentally regulated with negligible expression levels in day 3 mice, which is consistent with other reports that defensins are developmentally regulated [15, 16, 42].

Moreover, we observed the upregulation of CCL2 mRNA, also known as monocyte chemoattractant.

protein 1 (MCP-1), CXCL1, and CXCL2 in treated mice with the anti-CD71 antibody (Fig. 3H-J). Furthermore, we found the upregulation of IL-6 but downregulation of the peroxisome proliferator-activated receptor gamma (PPARγ) mRNA in the gut tissues of treated mice compared to controls, respectively (Fig. 3K,L). PPARγ acts as a sensor for hormones and metabolites with well-recognized anti-inflammatory properties [43]. Moreover, we noted that depletion of CECs was associated with the upregulation of TLR-4 and TLR-9 mRNA in intestinal tissues (Fig. 3M,N) without any changes in the expression levels of TLR-2, TLR-3, and TLR-5. Considering that CECs express arginase I, II, and TGF-β [30, 44], we found that their depletion significantly altered mRNA for TGF-β, Smad2, Smad3, and arginase I expression in the gut tissues of treated mice (Fig. 3O-R).

We also measured the expression of occludin (Ocln) and the tight junction protein 1 (Tjp1) genes,

which encode the tight junction-associated proteins. We found that the expression of both genes was significantly decreased in treated mice with the anti-CD71 antibody compared to controls (Fig. 3S,T), suggesting a possible role for CECs in maintaining gut integrity. This was further evaluated by measuring the intestinal permeability using oral administration of dextran conjugated with fluorescein isothiocyanate (FITC-dextran), as we reported elsewhere [27]. We observed that depletion of CECs increased the intestinal permeability of mice (3-day old) two-day post-treatment (Fig. 3U). Since the transferrin receptor (CD71) is also predominantly expressed on the basolateral surface of epithelial cells [45], we investigated the effect of the anti-CD71 antibody on the surface expression of CD71 using immunofluorescence staining. As shown in (Fig. 3V and S. Figure 5H), this antibody did not impact the expression level of CD71 in the gut tissues, supporting the notion that the anti-CD71 antibody depletes CECs without impacting CD71 expression in the gut as we have reported elsewhere [25]. Finally, we found that depleting CECs was associated with an increase in the cleaved caspase 3 (CC3), indicative of epithelial apoptosis (S. Figure 5I). Nevertheless, enhanced apoptosis was observed in conventional but not in germ-free animals (S. Figure 5I). This observation suggests enhanced apoptosis occurs in the presence of microbial communities and the absence of CECs but not due to the direct effects of the anti-CD71 antibody administration. To better delineate the relationship between microbiota and AMPs, we depleted CECs in germ-free mice and subsequently subjected them to SPF cohousing for 24 h before assessing the expression of mRNA for AMPs in their gut tissues. We found that depleting CECs in germ-free mice did not alter the expression of AMPs in their intestinal tissues compared to the control group (Isotype control treated) (Fig. 3W-Z). However, depletion of CECs in SPF cohoused neonates resulted in a significant reduction in the expression of CARMP, Mmp7, Mbd1, and Defα1 compared to their cohoused and untreated counterparts (Fig. 3W-Z). Of note, we observed a significant upregulation of CRAMP and Defα1 mRNA in gut tissues of germ-free mice when exposed to microbial communities (Fig. 3W, 3Z). Our observations contradict the notion that the anti-CD71 antibody has direct effects on the expression of AMPs.

## Modulating the frequency of CECs during the early stage of the neonatal period leads to changes in the gut microbial communities

To determine whether CECs influence the adaptation of early colonizers in the gut of newborns, we investigated changes in microbial communities once CECs were depleted. Considering that microbiomes cluster by cage, and to prevent confounders as we have reported elsewhere [46], treated and control pups were from the same litter and in a single cage. For these studies, we focused on the small intestine because it possesses the highest proportions of CECs (Fig. 1C,D). Moreover, it is reported that the composition of microbial communities is similar between the small intestine and colon in newborns [47]. Therefore, we collected small intestinal content from the anti-CD71 treated and control groups at day 4 (24 h post-treatment) and subjected them to 16S rRNA Illumina sequencing. UniFrac distance analysis identified distinct clustering of treated and control animals (Fig. 4A). Gut microbiota of all experimental animals was dominated by members of the phyla Proteobacteria and Firmicutes (Fig. 4B,C) and depletion of CECs at day 3 significantly affected relative abundance of these dominant bacterial families. The depletion of CECs in the early stage of life significantly increased microbial community richness calculated as observed by OTUs (Fig. 4D), Faith’s phylogenetic richness index, and Shannon’s diversity index and evenness (Fig. 4E,F). More importantly, the abundance of *Staphylococcus* and *Lactobacillus* genera was significantly increased among treated animals compared to their control counterparts (Fig. 4G,H). However, the abundance of *Escherichia* genus had the opposite pattern and was significantly decreased in treated animals (Fig. 4I), whereas, no significant difference was observed in the *Streptococcus* genus (Fig. 4J).

### The long-term effect of neonatal CECs on microbial communities is more dominant in females than in males

**Fig. 6:**
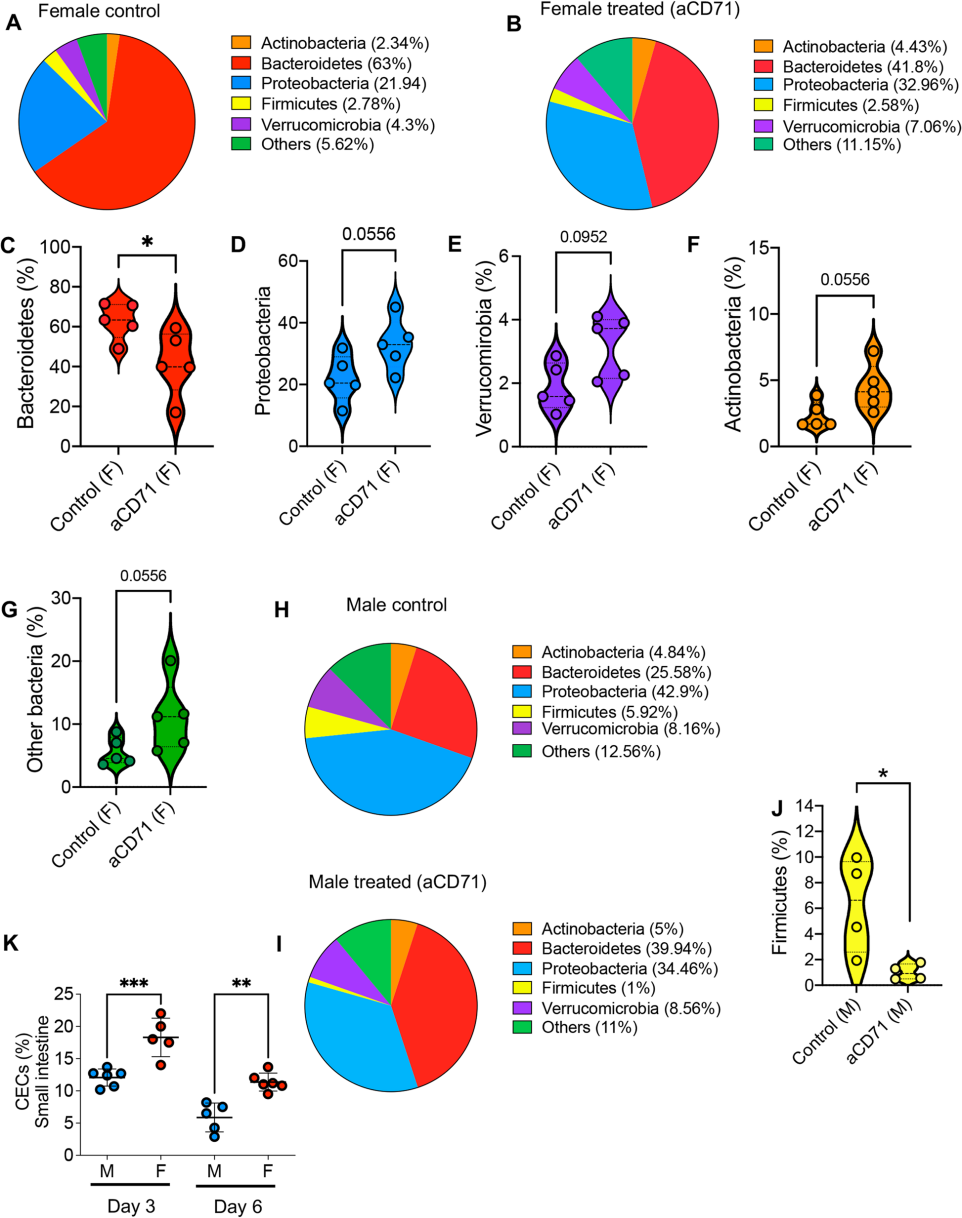
Depletion of CECs exhibits differential long-term effects on microbial communities in female vs male mice. **A** The pie chart plot depicts the abundance (%) of each main bacterial population in the control female, and **B** treated female mice with the anti-CD71 antibody 32 days later. **C** The abundance of *Bacteroidetes* bacteria, **D** *Proteobacteria*, **E** *Verrucomirobia*, **F** *Actinobacteria*, and **G** other bacteria in the small intestine of control female vs anti-CD71 treated female mice. **H** The pie chart plot depicts the abundance (%) of each main bacterial population in the control male, and **I** treated male mice with the anti-CD71 antibody 32 days later. **J** The abundance of *Firmicutes* bacteria in the small intestine of control vs treated mice with the anti-CD71 antibody 32 days later. **K** Cumulative data of the frequency of CECs in small intestine of day 3 and 6 male and female mice. *P* values were calculated using two tailed, Mann–Whitney *t* test (**C-G, J,K**). Results are presented as SD and (*P* < 0.05 (*), *P* ≤ 0.01 (**), *P* ≤ 0.001 (***). Anti-CD71 (aCD71). not significant (ns)

Although our observations showed that depletion of CECs results in alterations of the gut microbiota in neonatal mice, it was unclear whether such changes were transient or lasted to adulthood. Therefore, we depleted CECs in a subset of 3-day-old pups and the control group received the isotype antibody. To account for cage effects, littermates remained in the same cage at the weaning age (day 21), but their parents were removed. At age 35 days, we collected their intestinal content and subjected them to 16S rRNA gene Illumina sequencing. UniFrac distance analysis identified distinct clustering of control females and males (Fig. 5A). Using permutational multivariate analysis of variance (PERMANOVA) indicated a significant difference between the groups. Although treated animals were mainly segregated by sex, they exhibited a partial clustering compared to controls (Fig. 5A). The role of sex in shaping gut microbiome is well-documented in adult mice [48] but our results reveal such changes can be observed from a younger age. We found that the gut microbiota of all experimental animals was dominated by members of the phyla Proteobacteria, Bacteroidetes, Actinobacteria, Verrucomicrobia, and Firmicutes (Fig. 5B and S Fig. 6A). Furthermore, our analysis revealed that the microbial communities in the male mice not only demonstrated increased richness (Fig. 5C) but also exhibited greater diversity. This was evident through assessments using alpha evenness, Faith's phylogenetic richness, and Shannon's diversity indexes, when compared to the microbial communities in female mice (Fig. 5D-F). Next, we assessed microbial changes in the gut of male/female mice ~ one month post the depletion of CECs. We found that depletion of CECs in the early stage of life significantly increased microbial community richness OTUs (Fig. 5G), Alpha evenness index, and Shannon’s diversity index in female mice (Fig. 5H,I). However, despite a moderate increase Faith’s phylogenetic richness index was not significantly different in treated female mice versus their female siblings (Fig. 5J). Surprisingly, we did not find any significant difference between treated male mice versus the control group when similar analyses were performed (Fig. 5G-J). Although treated male mice clustered together (Fig. 5A), variability in control males may explain our results.

Our further analysis of bacterial communities revealed that the depletion of CECs was associated with a significant decrease in the abundance of Bacteroidetes in female mice (Fig. 6A-C). In contrast, the relative abundance of Proteobacteria, Verrucomicrobia, Actinobacteria, and other bacteria was increased among treated animals compared to their control counterparts even though such changes did not reach to a significant level (6A,B, and 6D-G). However, when similar analyses were performed among treated and control male mice, we only observed a significant decline in the abundance of Firmicutes bacteria (Fig. 6H-J). Moreover, we noted an increase and decrease trend in the abundance of Bacteroidetes and Proteobacteria in treated male mice versus control, respectively (Figs. 5I, 6H). These observations imply that modulation of CECs in the neonatal period can have long-lasting effects on the diversity and composition of gut bacterial communities. To understand to mechanism underlying sex difference effects, we quantified the frequency of CECs in the small intestine of male and female pups. Intriguingly, we observed that female pups had significantly higher frequency of CECs than their male counterparts (Fig. 6K). Therefore, our observations highlight the role of sex in the interplay between CECs and microbial communities.

### CECs are associated with high intestinal microbial counts and diversity during murine adulthood and their depletion during pregnancy causes microbial dysbiosis

**Fig. 7:**
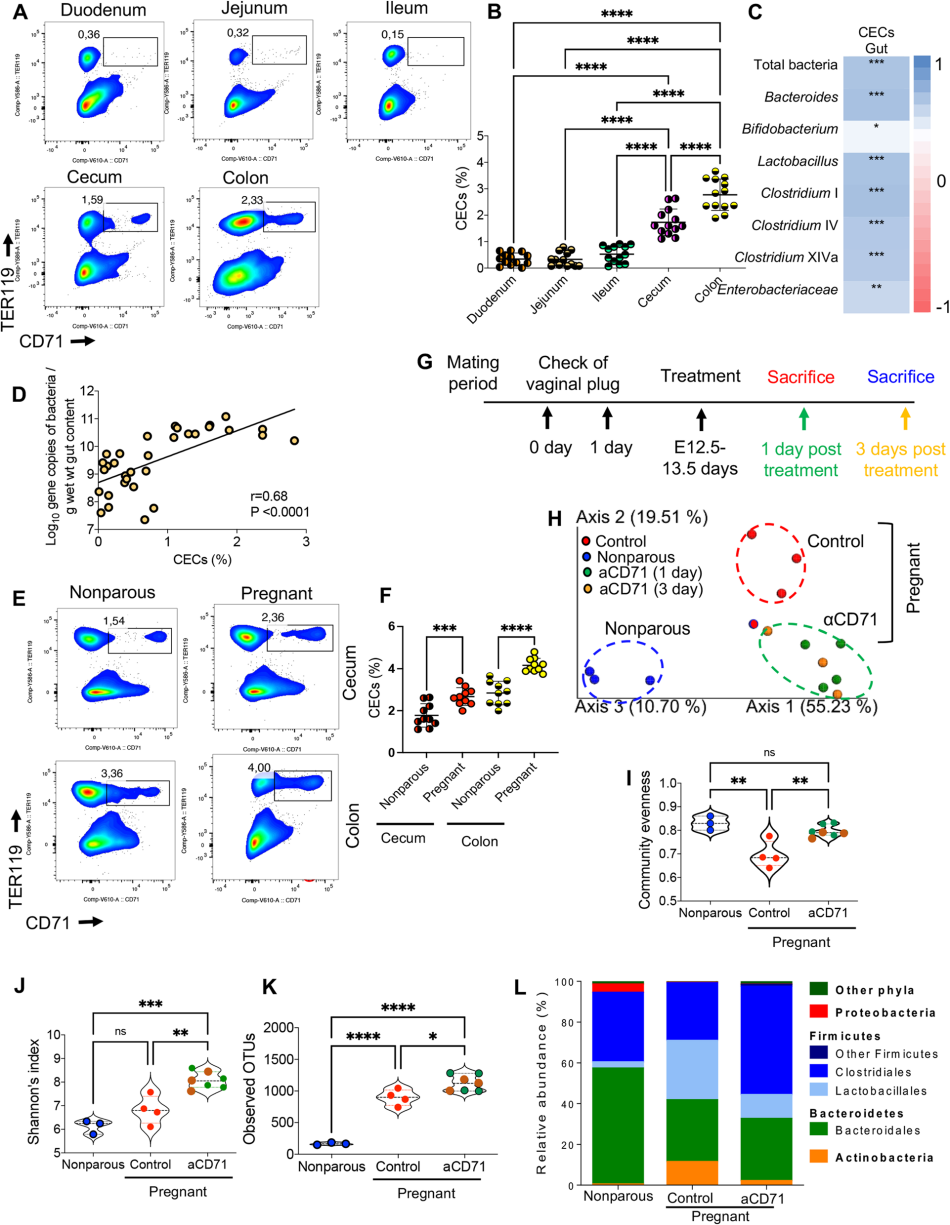
Association of CECs with the gut microbiota during adulthood and pregnancy. **A** Representative flow cytometry dot plots, and **B** Cumulative data showing the percentage of CECs in lamina propria of the duodenum, jejunum, ileum, cecum, and colon obtained from 10-week-old mice. **C** Heat map of Spearman’s correlation coefficients between copy numbers of dominant bacterial taxa assessed by qPCR and frequency of CECs in different parts of the gastrointestinal lamina propria of adult mice. **D** Data showing the correlation of the gut bacterial content quantified by qPCR with % of CECs in the full gut tissues. **E** Representative flow cytometry plots, and **F** Cumulative data depicting CEC proportions in the cecum and colon between pregnant and nonparous females. **G** Schematic representation of CECs depletion during pregnancy. Pregnant mice (E12.5-E13.5) were either injected with the anti-CD71 antibody or isotype control. Then spleen and gut samples were randomly collected from treated animals either one- or three-days post-treatment. Nonparous females, that has not given birth, were also included as control animals. **H** Principal Coordinates Analysis (PCoA) of weighted UniFrac matrix (beta diversity measure) identifying differences of microbial communities between control (pregnant and nonparous) animals and anti-CD71 treated mice. Each symbol represents an individual animal. Groups are color coded as follows: blue (nonparous group), red (pregnant control group), orange (1 day), and green (3 days after anti-CD71 treatment). **I** Alpha diversity measured by community evenness, **J** Shannon’s diversity index, and **K** observed OTUs. Symbols represent individual animals, and data are presented as median and interquartile ranges. **L** Relative abundance of dominant bacterial orders in colon samples obtained from nonparous, pregnant, and anti-CD71 treated pregnant mice. Results are presented as SD and *P* values were calculated using Kruskal–Wallis analysis with Dunn’s multiple comparisons test (B, F, I, J,K) or the Spearmen correlation analysis (D). *P < 0.05, **P < 0.01, *** *P* < 0.001, **** *P* < 0.0001

We have previously shown the expansion of CECs in the periphery of both humans and mice during pregnancy [26, 27]. However, this does not occur in the peripheral blood of pregnant mothers with IBD [27]. Notably, CECs from the cord blood and placenta of IBD mothers exhibit impaired effector functions [27], which was also the case in C-section-delivered twins [49]. Based on these observations we aimed to determine whether changes in the frequency of CECs during pregnancy can influence the composition of microbial communities or immune homeostasis in the gut. First, we quantified the proportions of CECs in the duodenum, jejunum, ileum, cecum, and colon of adult non-pregnant female mice. We found that the frequency of CECs was the highest in the colon and cecum compartments, which normally contain the highest number and more diverse microbial taxa (Fig. 7A,B). We quantified the density of bacteria in non-pregnant, pregnant (control and anti-CD71 treated) by real-time quantitative PCR (qPCR) using a wide range of bacterial primers (S. Table 1). Spearman’s correlation analysis was performed to determine any relationship between the frequency of CECs in intestinal tissues and bacterial taxa during adulthood. These analyses revealed a positive correlation between the total bacteria and dominant bacterial taxa with the intestinal proportion of CECs (Fig. 7C,D and S Fig. 6B, C). This correlation analysis involved pooling data from all gut compartments. Considering the variability in both CEC frequency and microbiota across these compartments, it is essential for future studies to determine the biological relevance of these observations or whether they are coincidental in nature. The reason behind this was to show that those gut compartments with low bacterial count and diversity have a lower percentage of CECs; while the cecum and colon, the gut compartments with the highest microbial count and diversity, have the highest percentage of CECs. Next, we quantified the proportions of CECs in the colon and cecum of nonparous versus pregnant mice. As shown in Fig. 7E,F, the proportion of CECs was significantly increased in the cecum and colon of pregnant compared to nonparous mice. To delineate the role of CECs on microbial communities during pregnancy, as shown in Fig. 7G, we treated pregnant mice with the anti-CD71 at E12.5–13.5 and quantified dominant bacterial taxa in their ileal, cecal, and colonic contents. Female animals were kept together and upon detection of pregnancy female animals were randomly allocated into control or treated to prevent the cage effect.

Quantification of dominant bacterial taxa using qPCR revealed changes related to pregnancy in the maternal microbiota in all three gut compartments (S Fig. 6D-F), which is reported to be associated with adjustment to the metabolic changes in pregnancy [50]. We observed that pregnancy resulted in a decrease in gene copies of members belonging to the *Enterobacteriaceae* family in different compartments of the gut. Additionally, counts of the *Bacteroides* group were lower in pregnant mice compared to nonparous females for cecum and colon, whereas, clostridial clusters I and IV were increased in pregnant mice (S Fig. 6D-F). Interestingly, we found that the depletion of CECs in pregnant mice resulted in significant changes in the gut microbiota. This impact was more evident in the colonic compared to the ileal and cecal microbiota (S Fig. 6D-F). These changes were associated with increased total bacteria, as well as increased gene copies of clostridial clusters IV and XIVa in treated animals versus controls (S Fig. 6D-F) Furthermore, colon contents were subjected to sequencing using 16S rRNA Illumina platform for a deeper insight in the role of CECs in microbial communities during pregnancy.

As illustrated in Fig. 7G, pregnant mice were subjected to analysis at 1- and 3-days post-treatment with the anti-CD71 antibody. For comparing colonic microbial communities, a weighted and unweighted UniFrac distance matrix was performed. The results of the weighted UniFrac analysis revealed separate clustering of the nonparous, pregnant control and the anti-CD71 antibody-treated pregnant groups (Fig. 7H). However, UniFrac distance analysis did not identify separate clustering between those two treated groups (Fig. 7H), therefore, we pooled all anti-CD71-treated animals together in one group for the remaining statistical analysis. Our analyses revealed that depletion of CECs was associated with increased microbial community evenness (Fig. 7I), Shannon’s diversity (Fig. 7J), as well as microbial richness (Fig. 7K) compared to control pregnant animals. These observations coincided with changes in the relative abundance of the colonic microbiota (Fig. 7L). Our results revealed a decrease in the Bacteroidetes phylum but an increased in the abundance of Firmicutes and Actinobacteria phyla in pregnant mice compared to non-pregnant females (Fig. 7L and S. Table 2). Moreover, we found a decrease in the abundance of *Bifidobacteriaceae* and *Lactolacillaceae* families, but in contrast, members belonging to the Clostridiales order such as *Lachnospiraceae*, *Ruminococcaceae,* and unclassified Clostridiales were increased in the absence of CECs compared to the control pregnant group (S. Table 2). Similar differences, characterized by a high abundance of the *Deferribacteraceae* family, Gram-negative bacteria performing anaerobic respiration using iron, were more evident among anti-CD71-treated pregnant females (S. Table 2). In accordance with the qPCR results, a relative abundance of members belonging to the Proteobacteria phylum, *Enterobacteriaceae,* and *Alcaligenaceae* families, were affected by the pregnancy, but not by the depletion of CECs (S. Table 2). Therefore, our observations provide a novel role for CECs in the maintenance of the gut microbiota not only in the neonatal period but also during pregnancy.

Moreover, to determine the impact of CEC depletion on immune homeostasis in the gut, we isolated ileum and colon tissues from different animal groups and subjected them to qPCR analysis. These studies revealed that similar to neonatal mice depletion of CECs resulted in upregulation of IL-6, CXCL-1, TLR-3, and TLR-4 but downregulation of TGF-β mRNA in colonic tissues (S. Figure 7). However, the expression levels of TLR-2 and TLR-9 remained unchanged between groups (S Fig. 7). Interestingly, we did not observe any significant difference in the expression levels of these analyzed genes in the ileal tissues. Theses differential compartmental effects might be explained by the abundance of CECs in the colon vs ileum.

### The decreased proportion of CECs in the cord blood of premature newborns may influence their microbial communities

Immunosuppressive CECs are highly abundant in human cord blood and placenta tissues [27, 51] and their frequency is reported to be lower in pre-term C-section deliveries [52]. Prematurity is associated with a lack of diversity in microbial communities, diminished gut barrier functions, and enhanced permeability [53, 54]. We decided to quantify the proportion of CECs in human cord blood at different gestation weeks. We observed significantly lower percentages of CECs in the cord blood of pre-term, < 34 gestation week, than full-term deliveries (S Fig. 8A-C). It appears that the proportion of CECs in the cord blood increases with the gestation age. While we were unable to obtain placental tissues from all gestational ages, CECs are abundant in placental tissues as previously reported [26] (S Fig. 8D). These observations imply that the physiological abundance of CECs in human cord blood/placenta may play an important role in immune homeostasis and symbiosis in the neonatal period. Although CECs are abundant in the peripheral blood of human neonates [22], their presence/role in the neonatal gut and interaction with microbial communities merits further investigations.

## Discussion

In this study, we show that CECs are highly abundant in intestinal tissues in the early stages of life but gradually decline with age. Although CECs are also abundant in the spleen of newborns, intestinal CECs possess a unique phenotype characterized by elevated expression levels of PD-L1, VISTA, ROS, and α4β7 integrin compared to their counterparts in the spleen. In particular, intestinal CECs consistently express higher levels of α4β7 integrin compared to their counterparts in the spleen. This integrin regulates lymphocyte migration to the intestinal lamina propria, which is mediated by its interaction with the vascular cell adhesion molecule MAdCAM-1 expressed by mucosal venules [55]. The gut microbiome plays an essential role in the induction of T regulatory 17 cells (Treg 17 cells), and the interaction of MAdCAM-1 and α4β7 is essential to retain these immunosuppressive cells in the gut [56]. Therefore, it is possible to hypothesize that the MAdCAM-1-α4β7 interaction may exert a similar function in retaining CECs in the gut. Notably, the presence of central macrophages and erythroblastic niches in the intestinal lamina propria of neonatal mice supports the existence of an extramedullary niche. Hence, CECs are not recruited but instead generated in intestinal tissues of newborns in a manner similar to hematopoietic stem and progenitor cells (HSPCs) [35]. This concept could be relevant in supporting their putative promotion of tolerance in the interface with microbial communities. This anatomical specialization of CECs might be part of the evolutionary mechanism or due to the maternal microbiota (e.g. microbial metabolites) that drives the development of CECs in the fetus/newborn as described for the innate immune system [57].

HSPCs [58] and early hematopoietic progenitor cells express functional TLRs and respond to TLR-ligands [29]. Nevertheless, the lack of central macrophages and subsequently hematopoiesis niches in intestinal tissues of germ-free mice suggests that pioneering microbes might interact with early HSPCs through their TLRs or other mechanisms to provoke extramedullary erythropoiesis in the gut [59]. If the fetus is sterile in utero, [11, 60] there should be no direct microbial signals inducting CECs in the fetal gut. Thus, we suggest that the maternal microbiome and/or its by-products may drive this process of development [57]. However, further investigations are required to establish the molecular/ cellular mechanics and the timing of this process in response to microbial colonization in the neonatal gut.

The physical barrier of the mucus layer and the cellular components, including the gut-associated lymphoid tissue (GALT) and the intestinal epithelial cells (IEC), play a crucial role in maintaining a symbiotic relationship with the commensal microbes [61]. Disturbance of this relationship could provide unrestrained access of normal gut residents or their by-products to the lamina propria, leading to an inflammatory response [62]. In line with this, we observed an intensifying pro-inflammatory response in the gut characterized by enhanced TNF-α secretion by the residential myeloid cells but reduction of intestinal Tregs following the depletion of CECs. Therefore, intestinal CECs may protect against excessive inflammation triggered by commensal microbes in the neonate.

Furthermore, microbial sensing plays an essential role in regulation AMPs and maintaining tight junction integrity [63, 64]. Consequently, our studies revealed that the depletion of intestinal CECs led to a significant alteration in the expression in a wide range of genes associated with immune homeostasis and the integrity of the intestinal epithelial barrier. In particular, the depletion of CECs in younger mice led to the downregulation of genes encoding AMPs. Since Paneth cells and Paneth-cell-derived AMPs are absent in neonatal mice and typically appear approximately 2 weeks after birth [65], the primary source of innate host defense effector molecules is the IECs before the formation of Paneth cells [66]. Of note, we observed that CECs-mediated alteration in AMPs was microbiome dependent. Because the anti-CD71 treatment did not influence the expression of these innate immune peptides in germ-free animals but it observed when treated mice were cohoused in SPF condition.

Consequently, the modulation of CECs could favorably alter microbial dysbiosis. This was further supported by substantial alterations in the frequency and diversity of microbial communities. Likewise, the association between CECs and changes in the microbiota in preterm infants with Bronchopulmonary dysplasia (BPD) [67], supports the notion that CECs may influence bacterial biodiversity and subsequently impact immune regulation in the gut. These observations underscore the crucial role of CECs in symbiosis during the neonatal period, which may have long-lasting effects. In line with this, we found that modulation of CECs in the young can result in dysbiosis that persists into adulthood. Nevertheless, the long-term CEC-mediated dysbiosis was more prominent in female mice than in males. Although the mechanism underlying these observations merits further investigations, we noted a higher proportion of CECs in intestinal tissues of female juvenile than male mice as reported in adult females [68]. The occurrence of earlier puberty in females and subsequently estrogen effects on increase in proliferation of hematopoietic stem cells (HSC) in females/pregnancy [69]may contribute to sex-related disparities in CECs and microbial communities.

Moreover, given the expansion of CECs during pregnancy [26, 27], we observed the cecal and colonic lamina propria of adult mice exhibit the highest frequency of CECs, aligning with the reported greater quantity of bacterial species in these microenvironments [70]. Interestingly, we found the expansion of cecal and colonic CECs during pregnancy coinciding with changes in the host-microbial interface [71]. The immune, hormonal, and metabolic changes that occur during pregnancy have a notable impact on the gut function, consequently modulating bacterial composition [50, 72]. These shifts in maternal microbial composition facilitate the necessary adjustments in maternal metabolism to support pregnancy and optimize the vertical transmission of maternal bacteria during vaginal delivery [50]. Moreover, during the late stages of pregnancy, we observe microbial diversity and composition in the gut characterized by the abundance of Firmicutes but low presence of Bacteroidetes. Notably, we observed similar bacterial changes in pregnant mice compared to nonparous controls. Specifically, clostridial clusters IV and XIVa, from Firmicutes phylum increased, while bacteria belonging to the *Bacteroides* group and *Enterobacteriaceae* family were decreased.

However, the depletion of CECs during the late stage of pregnancy leads to a shift in gut microbial composition. This implies that CECs may play an instrumental role in modulating immune response to microbial colonization, even during pregnancy. Subsequently, any changes in their proportion and functionality can intensify the immune response to colonization with microbial communities, potentially resulting in dysbiosis early in life and during pregnancy. While the practical modulation of CECs in human neonates is challenging, the lower frequency of CECs in the cord blood of pre-term deliveries [49, 52] might explain dysbiosis and other immune-mediated dysregulations in premature babies [53, 73]. Although the exact molecular mechanism(s) of immunosuppression and immunomodulation of CEC in intestinal tissues remains elusive, we believe that CECs mediate their immunoregulatory functions via cell-to-cell interactions (e.g. PDL-1:PD-1 and VISTA) or soluble factors (arginase-I, arginase-II, ROS, and TGF-β) [31]. To truly dissect the mechanisms by which CECs mediate, potentiate, or respond to the microbiome and how these interactions impact microbial communities, further in-depth studies are warranted. The complexity of gut cellular biology necessities the development of interdisciplinary approaches to gain a better understanding of the dialogue between the microbiota and the immune system.

We are aware of multiple study limitations. Firstly, we acknowledge a limitation in our ability to investigate how the use of the anti-CD71 antibody affects microbial changes in mice beyond 5 weeks of age due to practical constraints. Secondly, there's a possibility that the anti-CD71 antibody could deplete activated immune cells expressing CD71. However, under steady conditions the frequency of CD71 + immune cells is very low to negligible levels.

Thirdly, we noted that while the depletion of CECs in neonatal mice led to an increase in *Lactobacilli*, this effect was not observed in pregnant mice. The reason for this difference is unclear but microbial communities in adults and newborns are completely different.

Additionally, the reduction in CECs in cord blood may not accurately reflect what's happening in the gut. This raises questions about the presence of erythropoiesis niches in human newborns, which requires further investigations. Although we were unable to evaluate the effects of CECs re-establishment on microbial communities, such studies are needed to better understand the cross-talk between CECs and the microbiome. Finally, we were unable to perform single cell RNA sequencing (ssRNAseq) on splenic versus intestinal CECs to better characterize their functional properties. Nevertheless, our study highlights the need for further investigation to determine whether the changes in microbial communities are directly linked to the effects of CECs or if they are indirect, possibly mediated through alterations in the immune response.

## Materials and methods

### Animals

Conventional BALB/c mice were used for this study. Mice were maintained and bred in a specific pathogen-free environment within the animal care facility at the University of Alberta. Mice were bred in house and pregnancies were checked twice daily to determine pregnancy timing. To prevent cage effects on the microbiota we made groups of mice cluster by cage. For neonatal studies, littermates were randomly divided into control and treated groups. Similarly, approach was used for pregnancy studies and non-pregnant adult mice. Germ-free mice were housed in tightly controlled and monitored isolators, and checked on a regular basis to confirm the germ-free status.

### In vivo studies

For the in vivo depletion of CECs, 3-day-old newborn mice were injected intraperitoneally (i.p.) with either 150 µg purified anti-CD71 (8D3) or the isotype control antibody (Rat IgG2a antibody) as we have reported elsewhere [22–24]. All newborn mice were euthanized one day after the anti-CD71 treatment, and the depletion of CECs was confirmed by measuring the percentages of CECs in the spleen and gut samples by flow cytometry. For long-term studies, 3-day-old newborn mice were treated with the anti-CD71 antibody as described above and kept in a single cage until day 35.

To study the recruitment of CECs to the gut, 3-day-old neonatal mice were either injected (i.p.) with 150 μg anti-α4β7 (DATK32; InVivoMab) and controls received the isotype control antibody. All experimental animals were euthanized at 2-days after treatment, and spleen and gut samples were harvested for further analyses.

### Timed pregnancies

BALB/c mice were paired, and females were checked for vaginal plugs to confirm their mating. The first day of gestation was considered to be the day after the plug was observed. On embryonic days E12.5-E-13.5, experimental animals were injected (i.p.) with either 200 µg purified anti-CD71 (8D3) or the isotype control antibody. One day after the treatment, some animals were randomly selected and euthanized and the rest of treated females were sacrificed three days later. A second control group, consisted of nonparous adult females, was also included in the study. The spleen, cecal, colonic tissues and their contents were collected at indicated time points for flow cytometry, as well as for the microbiota analysis. It is worth mentioning that under steady physiological conditions, the expression of CECs is negligible on other immune cells. Therefore, we do not expect any adverse effects of the anti-CD71 antibody on other immune and gut cells. However, under pathological conditions when immune cells become activated, such as T cells, we observe an upregulation of CD71. Therefore, these potential side effects should be taken into consideration in cancer models and other pathological conditions. Furthermore, based on our in vitro studies, this antibody does not have any direct effects on the viability of different bacterial communities.

### Sample processing

Single-cell suspensions were obtained by grinding spleen and liver samples between sterile frosted glass slides in 7 ml red blood cell (RBC) lysis buffer according to our previous methods [23, 25]. To obtain single-cell suspensions of lamina propria lymphocytes, the small intestinal, cecal and colonic tissues were collected and processed as we have described elsewhere [23]. Also, the bone marrow (BM) was flushed out from tibiae and femurs collected from BALB/c mice by using insulin syringes.

### Flow cytometry

Antibodies specific to mouse cell surface antigens were obtained from the Thermo Fisher Scientific or BD Biosciences, and targeted the following surface markers: anti-CD71 (C2), anti-TER119 (TER-119), anti-CD3 (145-2C11), anti-CD4 (GK1.5), anti-CD8a (53–6.7), anti-CD11b (M1/70), anti-CD11c (HL3), anti-CD69 (H1.2F3), anti-VISTA (MIH65), anti-PD-L1 (MIH5), anti-α4β7 (DATK32), anti-F4/80 (6F12), anti-CD169 (Siglec-1, 3D6.112), anti-VCAM-1 (51-10C9), anti-TNF-α (MP6-XT22), and anti-IFN-γ (XMG1.2). Live/dead dye was used for excluding dead cells (Thermo Fisher Scientific). Cells obtained from different tissues were stained in PBS with 2% FBS for 30 min at 4ºC in the dark and then fixed in 4% paraformaldehyde. Samples were acquired on a LSRFORTESSA flow cytometer (BD Biosciences), and data were analyzed using FlowJo (version 10, FlowJo LLC). For intracellular cytokine staining (ICS), isolated cells from the digested gut tissues were cultured ex-vivo in the presence of Glogi-blocker for 5 h prior to staining as we reported elsewhere [25, 74].

### Measuring reactive oxygen species

To detect intracellular generation of reactive oxygen species (ROS), 2’,7’-dichlorofluorescein diacetate (DCFH_2_‑DA, Sigma) was used according to the manufacturer’s protocol, and DCF fluorescence was measured by flow cytometry [75].

### Intestinal permeability assay

Two days after anti-CD71 treatment, neonatal mice were fed with 40 mg per 100 g body weight of fluorescein-isothiocyanate-dextran (FITC-dextran; Sigma-Aldrich) according to our previous report [25]. Mice were euthanized 4 h later and blood samples were obtained by cardiac puncture and the concentration of FITC-dextran in serum samples was assessed by measuring fluorescence intensity in a plate reader at 488 nm and dilutions of FITC-dextran in PBS were used for the standard curve.

### Immunofluorescence (IF)

Small intestinal tissues were collected 1–2 days after treatment with the anti-CD71 antibody or isotype control. Tissues were fixed in 4% para formaldehyde and stored overnight at room temperature. Then, cross-sectioned organs were embedded in paraffin using Leica TP 1020 tissue processor, and paraffin blocks were sectioned at 5-µm thickness employing Leica RM2255 microtome. Sections were transferred to slides, deparaffinized and rehydrated, followed by blocking of slides for 1 h in 10% serum of secondary antibody host. The anti-F4/80 (Abcam, ab300421) and biotin rat anti-mouse TER119 (BD Bioscience, Cat#553,672) were used as primary antibodies, staining was performed in humid chamber overnight in the dark. After this incubation step, slides were washed 3 times with TBS and once with PBS to reduce background. Then, secondary antibodies (Alexa Flour 488 Donkey anti rabbit (Thermo Fisher, A32790) and Alexa Fluor™ 555 streptavidin (Thermo Fisher, S32355) were added and slides were incubated for 1 h at room temperature in the dark, followed by washing step with TBS and PBS and the fluorescent DNA stain DAPI (4′,6-diamidino-2-phenylindole) was added for 15 min. After the last washing step, slides were mounted with 40 μL of Prolong Gold Antifade Reagent *(*Fisher Scientific, P10144) and dried overnight, as we have reported elsewhere [76]. Imaging was performed using Leica THUNDER microscope and a Zeiss 710 confocal. To determine the intestinal epithelial cells (IEC) proliferation, the deparaffinized and rehydrated tissue sections were also stained with anti-cleaved caspase 3 (CC3). CC3-positive epithelial cells were counted within 10 micrograph fields (original magnification × 100) per mouse. Tissue samples obtained from the anti-CD71antibody treated and control animals.

### Gene expression assay

Total RNA was extracted from different tissues in TRIzol (Invitrogen) using the RNeasy Mini Kit (Qiagen). Prior to extraction, tissues were homogenized by bead-beating with 2.8 mm stainless steel beads (Precellys) on a FastPrep-24 (MP Biomedicals) in 3 cycles of 20 s at 4 m/s speed. QuantiTect Reverse Transcription kit (Qiagen) was used to convert 1 μg RNA to cDNA, which was then subjected to quantitative PCR (qPCR). TaqMan PCR assays for each targeted gene were run in duplicates on CFX96 TouchTM Real-Time PCR Detection System (BioRad) (S Table 2). Fold change in gene expression was calculated relative to control groups using the 2^−ΔΔCt^ method, where β-actin was used as a housekeeping gene.

### Bacterial quantification by qPCR

Bacterial genomic DNA was isolated from gut contents using a QIAamp DNA Stool Mini Kit (Qiagen). To increase the recovery of bacteria DNA, particularly from Gram-positive bacteria, a bead-beating step with 0.1 mm Zirconium beads was also included to physically disrupt bacterial cells. Concentration and quality of DNA samples were checked on Nano-Drop ND-1000 Spectrophotometer (NanoDrop Technologies), and samples were diluted to a final concentration of 20 ng/µl to obtain comparable DNA amount.

Bacterial quantification was performed on CFX96 Touch™ Real-Time PCR Detection System (BioRad) using QuantiFast SYBR Green PCR master mix (Qiagen). Group-specific primers and qPCR conditions were as previously described [27]. qPCR products were verified by melting curves, obtained by a stepwise increase of the temperature from 60 to 95ºC (at 10 s/0.5ºC). The standard curves of the individual qPCR assays were constructed using PCR products generated with the same groups-specific primers and genomic DNA isolated from colonic contents as a template. Ten-fold serial dilutions of purified and quantified PCR products were used to generate the standard curves.

### Illumina MiSeq sequencing and data analysis

V4 variable region of 16S rRNA gene was amplified from the genomic DNA samples, and 250-bp paired-end sequencing was performed at the McGill University and Génome Québec Innovation Centre (Montreal, QC, Canada). Sequencing data delivered by the Innovation Centre were already demultiplexed and binned into individual samples according to their barcodes. Further bioinformatic analysis was performed using the QIIME2 pipeline (2018.11 release). The first step of this analysis was to join the paired-end reads (1 and 2) with a minimum of 100 bp overlap and 0 mismatches. Reads were then quality filtered by removing sequences having more than 10 sites with a Phred quality score less than 20. Next, reads were denoised into amplicon sequence variants (ASVs) using Deblur method. Taxonomy classification at the phylum, family and genus levels were done by comparing ASVs to the Green genes bacterial reference database (v. 13.8). Diversity indices (Evenness, Observed OTUs, Shannon’s diversity and Faith’s phylogenetic index) and distances between samples (weighted- and unweighted-Unifrac) were all calculated in QIIME2 to profile gut microbiota in individual luminal samples. Prior to taxonomy classification and generating alpha and beta diversity metrics, data were rarefied across samples for normalization such that all samples have the same number of total reads. Chimera removal was included in the data processing. The data for 4-day old pups were rarefied to a sequence depth of 16,149, pregnant mice date was rarefied at 30,660 sequence depth, while data from female/male older pups was rarefied at 13,491 sequence depth.

Raw data was deposited in the SRA database of NCBI and is publicly available under accession number PRJNA986906, PRJNA986955, and PRJNA986959.

### Statistics

Data analysis was performed with the Statistical Analysis Systems (SAS) Software (version 9.4; SAS Institute Inc.), and graphical presentation was done using GraphPad Prism (version 6.00; GraphPad Software). The Wilks-Shapiro test assessed the distribution of data. The Mann–Whitney U-test used for non-normally distributed data whereas one-way ANOVA followed by Tukey’s test was used when more than two groups were compared. Results were presented with standard deviation and significance set at *P* value < 0.05. The non-parametric Wilcoxon rank sum test or the Kruskal–Wallis test for > 2 group comparisons were used for not normally distributed variables. Spearman’s correlation test was also applied to test for any relationship between microbial taxa and host CECs using GraphPad Prism.

### Supplementary Information

Supplementary Material 1: Supplementary Figures S Fig 1. A) The gating strategy for the identification of CECs in intestinal tissues. B) Representative flow plots and cumulative data of the % of CECs in the spleen, small intestines, and colon tissues of 4-day-old control mice vs those treated with the anti-CD71 antibody a day earlier.  C) Gene expression assay for detection of α4β7 integrin, and D) its ligand MAdCAM-1 in small intestinal tissues of control or CECs depleted animals. E) Correlation analysis of % CECs with the intensity of α4β7 in splenic CECs in 3-day-old mice.  F)  Representative flow cytometry plots showing the gating strategy for central macrophages (CD11b-CD169+F4-80+) in the small intestine of a 3-day-old mouse. G) Representative flow plots of central macrophages in a 3-day-old germ-free mouse. Results are presented as SD and *P* values were calculated using two tailed, Mann–Whitney *t* test (B-D) or the Spearmen correlation analysis (E).  *P* < 0.05 (*). Anti-CD71 (aCD71). not significant (ns). S Fig. 2. Representative immunofluorescence staining (IF) plots at different indicated magnifications illustrating the presence of erythropoiesis niches in submucosal tissues of small intestine. DAPI (blue), TER119 (red), F4/80 (green). S Fig. 3. Representative IF plots at different indicated magnifications illustrating the presence of erythropoiesis niches in the villus of small intestine. DAPI (blue), TER119 (red), F4/80 (green). S Fig. 4. A) Representative IF plots illustrating the presence of mature red blood cells, without nuclei, in venules of small intestine. B) Representative IF plot illustrating the presence of erythropoiesis niches in the spleen of a neonatal mouse. DAPI (blue), TER119 (red), F4/80 (green). S Fig. 5. A) Representative flow cytometry plot of central macrophages in a germ-free mouse. B) Representative histogram and cumulative data of α4β7 expression in intestinal CECs of 3-day old conventional and germ-free mice. C) Representative flow cytometry plots, and D) cumulative data of % of CECs in the small intestine of conventional (con.) vs. germ-free mice. E) Representative flow cytometry plots, and F) cumulative data showing TNF-α production by intestinal CD11b+ and CD11c+ cells among treated mice (3-day old) with the anti-CD71 antibody or the isotype control antibody 24 hr later quantified by intracellular cytokine staining *ex-vivo*. G) Cumulative data of fold change in TNF-a expression in either CD11b+ or CD11c+ cells from germ-free mice treated with the anti-CD71 antibody compared to control animals. H) Quantified expression levels of CD71 obtained from the anti-CD71 treated and control mice (2-day post-treatment). I) Comparison of the mean number of cleaved caspase-3-positive (CC3+) intestinal epithelial cells (CC3+ IEC) per high power field (HPF) between the anti-CD71-treated versus isotype control in conventional or germ-free mice (treated at age 3 and examined at day 5). The immunofluorescence staining for CC3 was used as a marker of apoptosis and the experiment was performed with 10 mice per group with 10 fields per mouse. Results are presented as SD and*P* values were calculated using two tailed, Mann–Whitney *t* test (B, F-H) and One-way ANOVA (D, I).*P* < 0.05 (*), **P < 0.01, *** *P* <0.001, **** *P* < 0.0001. Anti-CD71 (aCD71), not significant (ns). S Fig. 6. A) Showing the relative frequency of dominant bacterial communities at the phylum level in control female (Con. F), control male (Con. M), or anti-CD71-treated (male (M) or female (F) mice. Mice were treated at day-3 and their gut contents were collected at day 35. B) The qPCR data showing the correlation between total gut CECs (%) with the gene copies of Lactobacillus or C) the Enterobacteriaceae family in the gut of adult mice. D) Cumulative data of the gene copies of bacterial taxa in nonporous vs pregnant mice (controls or treated with the anti-CD71) in the cecum, E) Ileum, and F) Colon. Results are presented as SD and *P* values were calculated using One-way ANOVA (D-F). (*P* < 0.05 (*), P £  0.01 (**), and P £ 0.00001 (****). Anti-CD71 (aCD71). S. Fig. 7. Cumulative data showing fold regulation of different indicated genes as quantified by qPCR in the colonic tissues of non-pregnant, pregnant control, and pregnant mice treated with the anti-CD71 at E12.5-13.5 and collected one day later. Each dot represents an animal. Results are presented as SD and *P* values were calculated using Kruskal–Wallis analysis with Dunn’s multiple comparisons test. (*P* < 0.05 (*), P £  0.01 (**), and P £ 0.0001 (***). Anti-CD71 (aCD71). not significant (ns). S Fig 8. A) Representative flow cytometry plots of the gating strategy for the cord blood CECs. B) Representative flow cytometry plots, and C) cumulative data of percentages of CECs in different gestational ages as indicated compared to an adult peripheral blood. D) Representative flow cytometry plot of CECs in a placental tissue from a full-term delivery. Each dot represents a cord blood. Results are presented as SD and *P* values were calculated using One-way ANOVA test. P £  0.01 (**), and P £ 0.00001 (****), not significant (ns).

Supplementary Material 2: S Table 1

Supplementary Material 3: S Table 2

## Data Availability

All data generated or analyzed during this study are included in main and supplemental materials. Raw data was deposited in the SRA database of NCBI and is publicly available under accession # PRJNA986906: https://dataview.ncbi.nlm.nih.gov/object/PRJNA986906?reviewer=m39a2g1tl0uc0e9if5pieplmdm PRJNA986955: https://dataview.ncbi.nlm.nih.gov/object/PRJNA986955?reviewer=n5ro73ure373vpivf95itunh0o And PRJNA986959: https://dataview.ncbi.nlm.nih.gov/object/PRJNA986959?reviewer=b4ogpnu54h4ghem7ffca7trad0
