# Supplementary figures and images for: CD71 + erythroid cells promote intestinal symbiotic microbial communities in pregnancy and neonatal period
Source: Microbiome. 2024 Jul 30;12:142. doi: 10.1186/s40168-024-01859-0 (PMC11290123; doi:10.1186/s40168-024-01859-0)

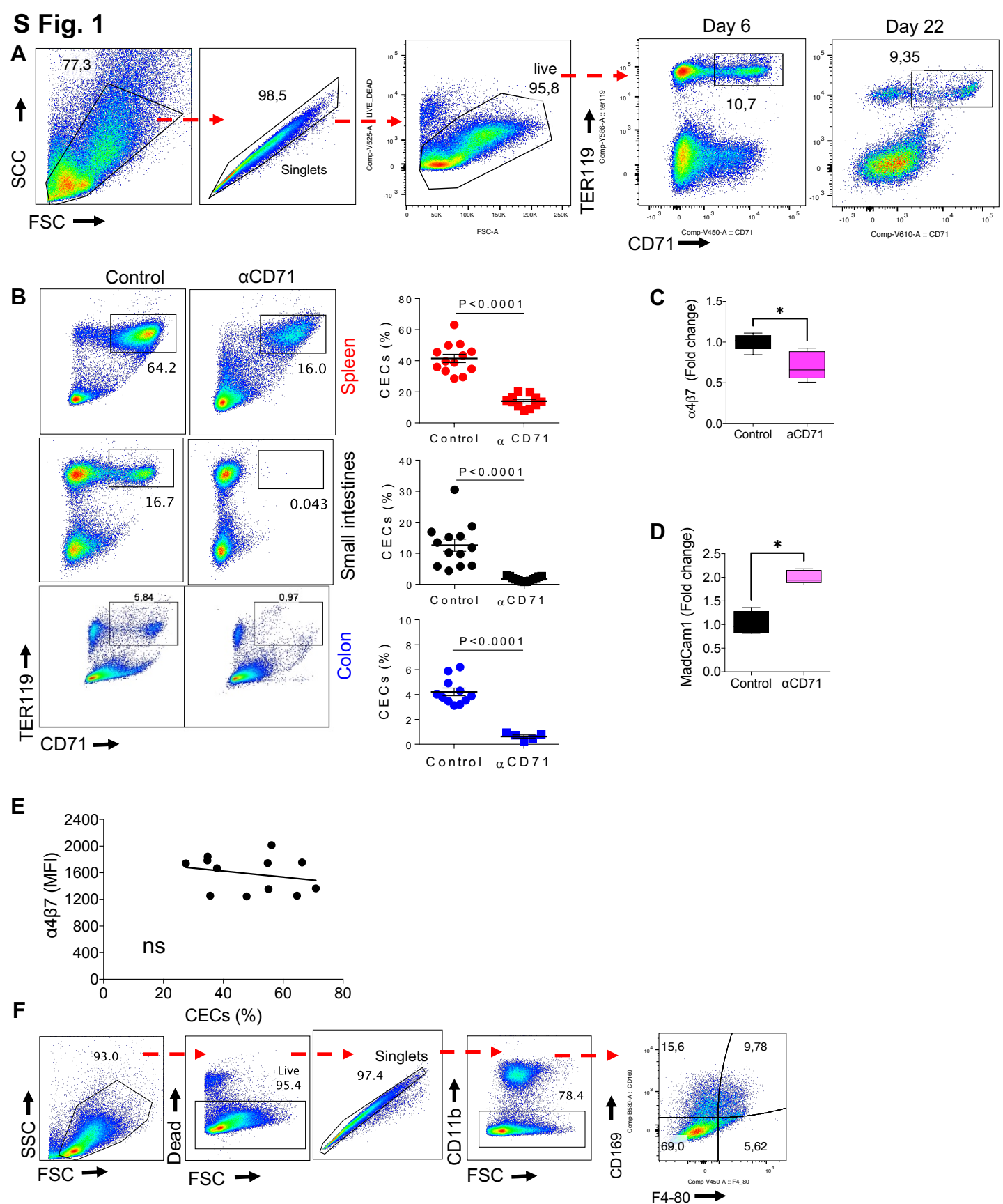

S Fig. 2

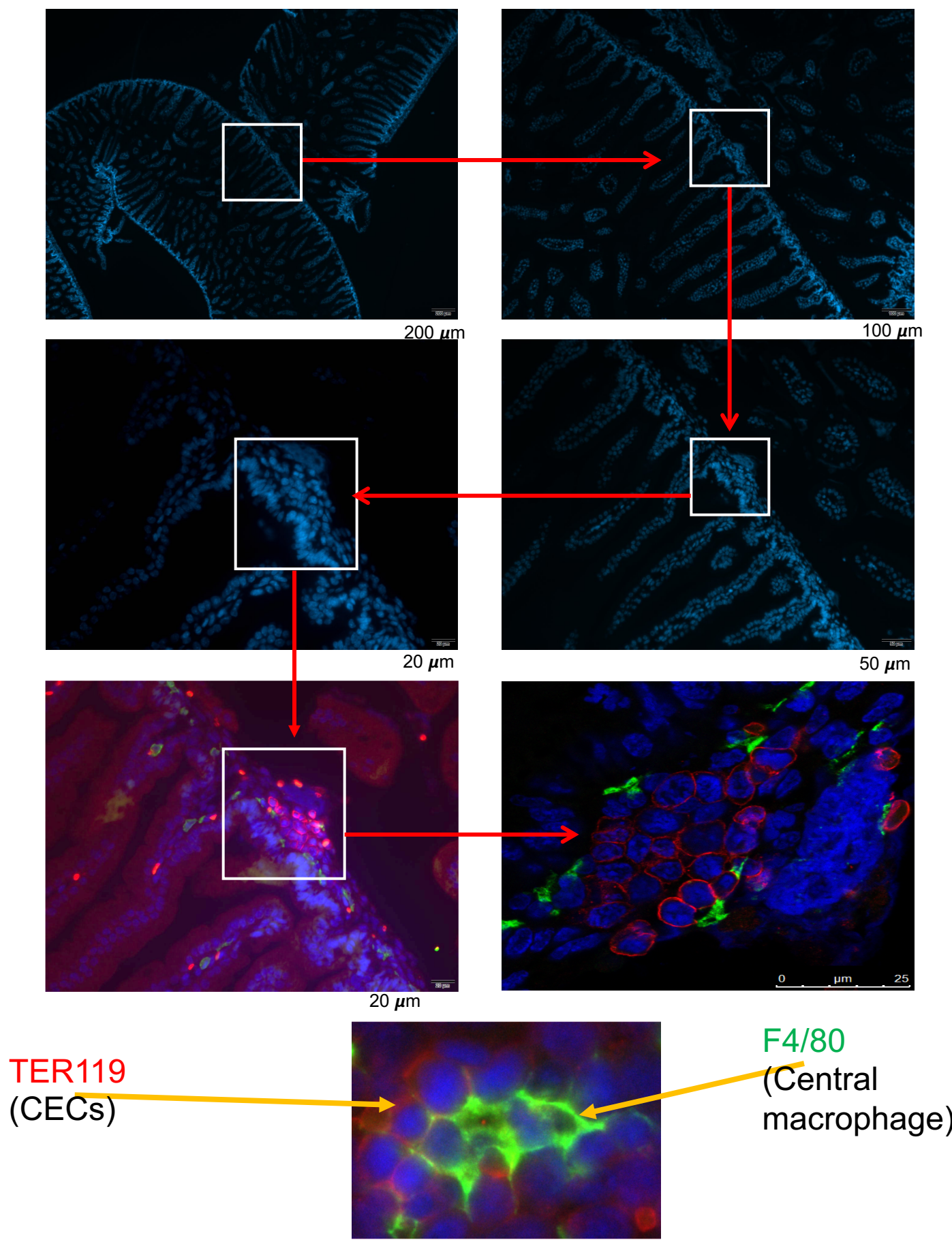

S Fig. 3

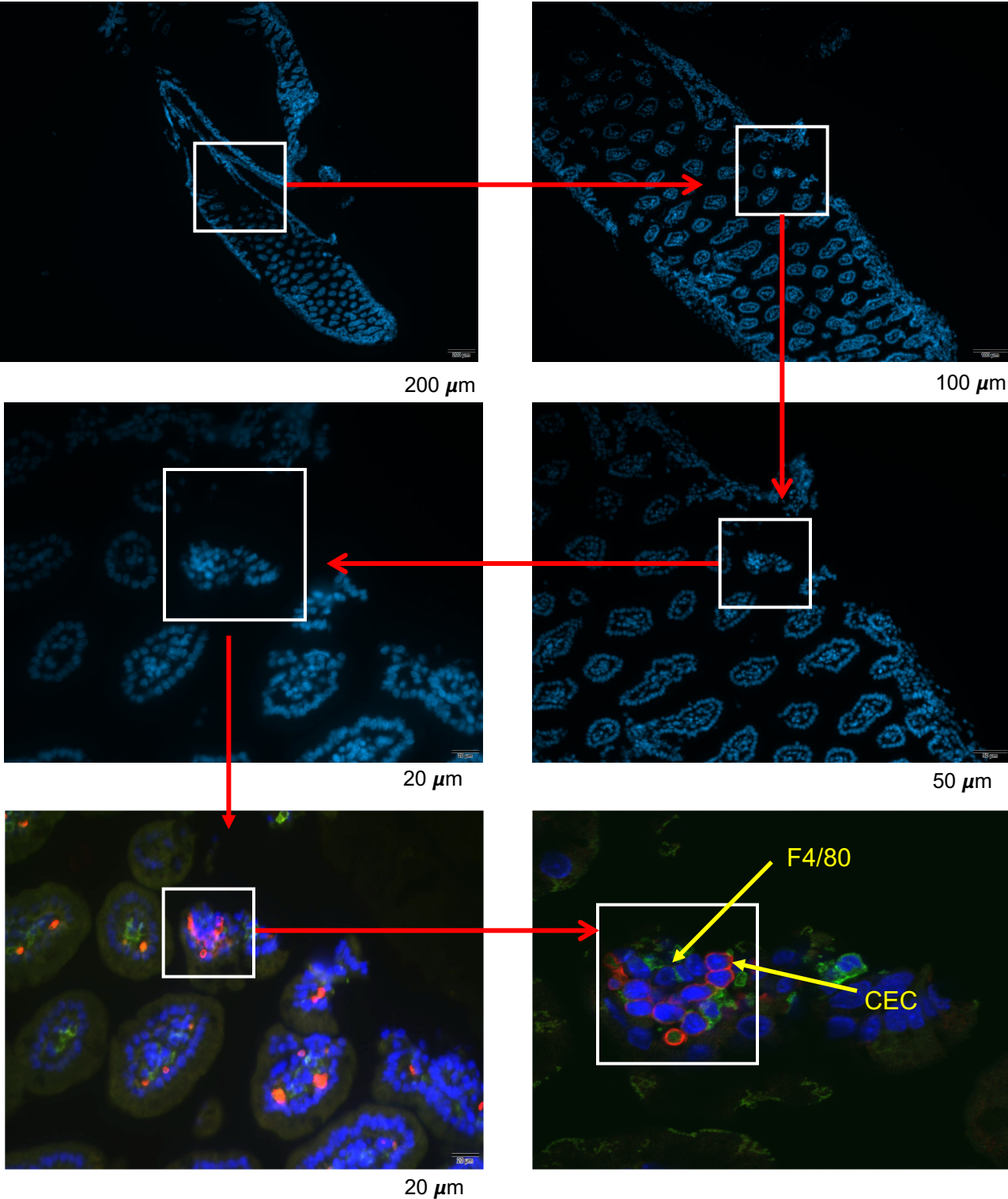

S Fig. 4

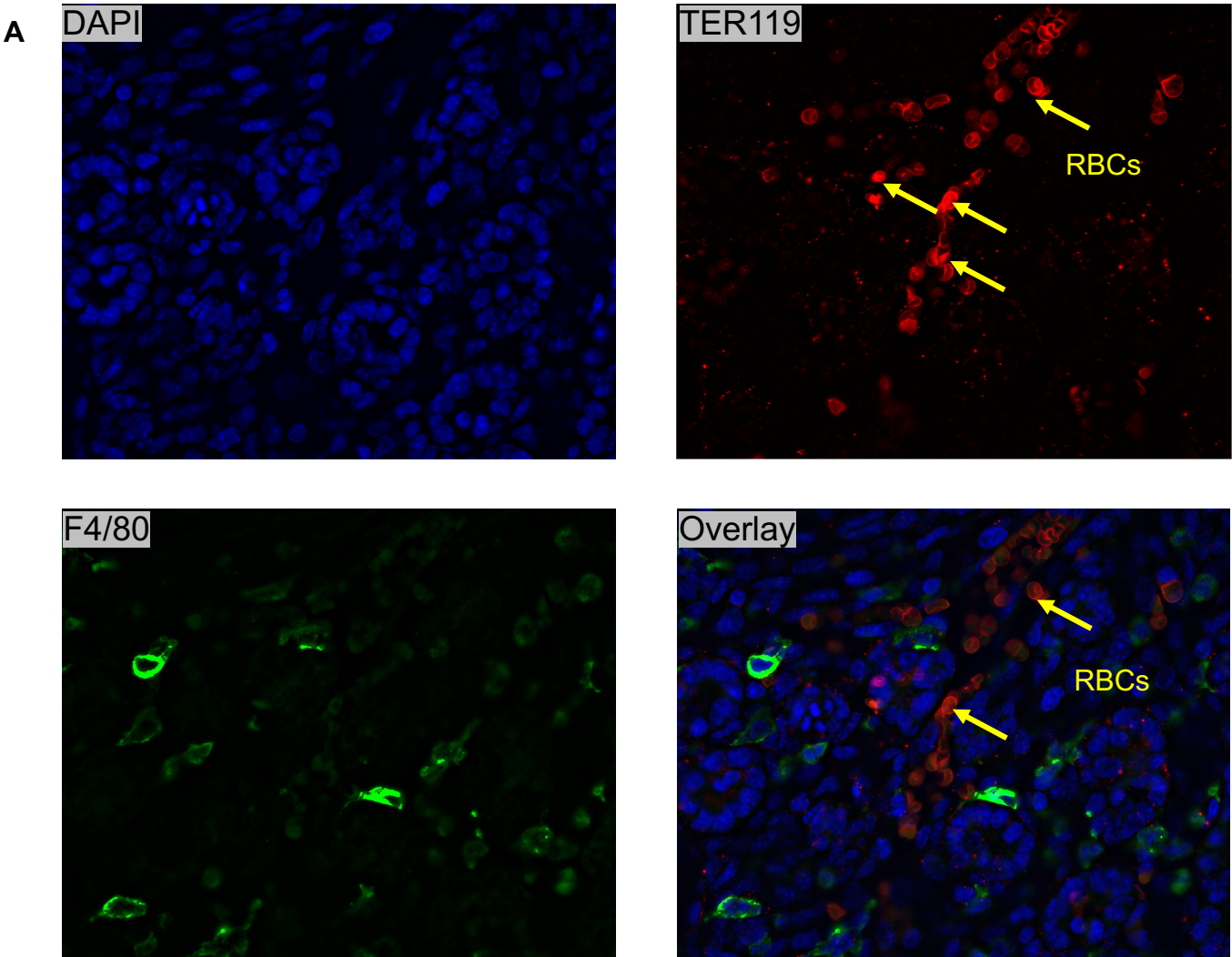

**B**

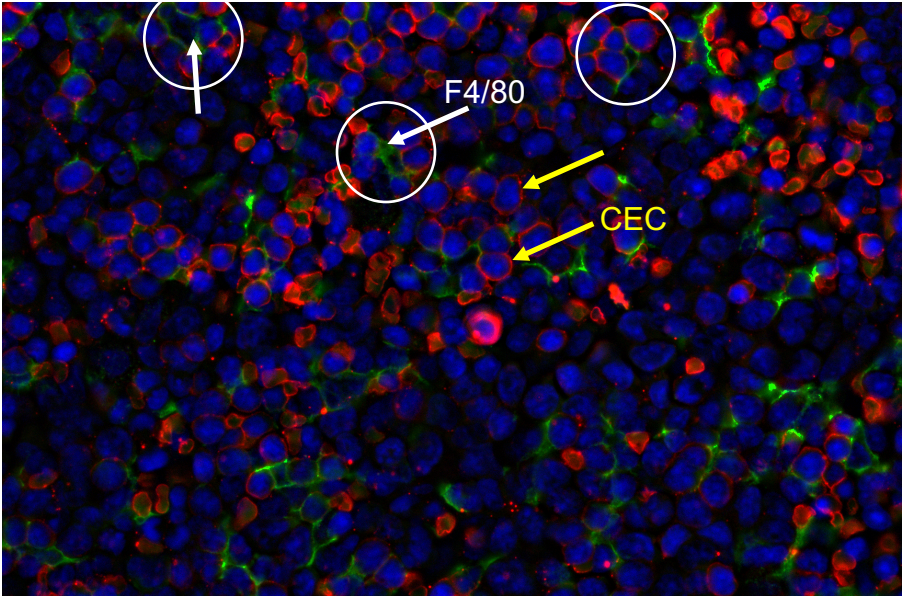

**S Fig. 5**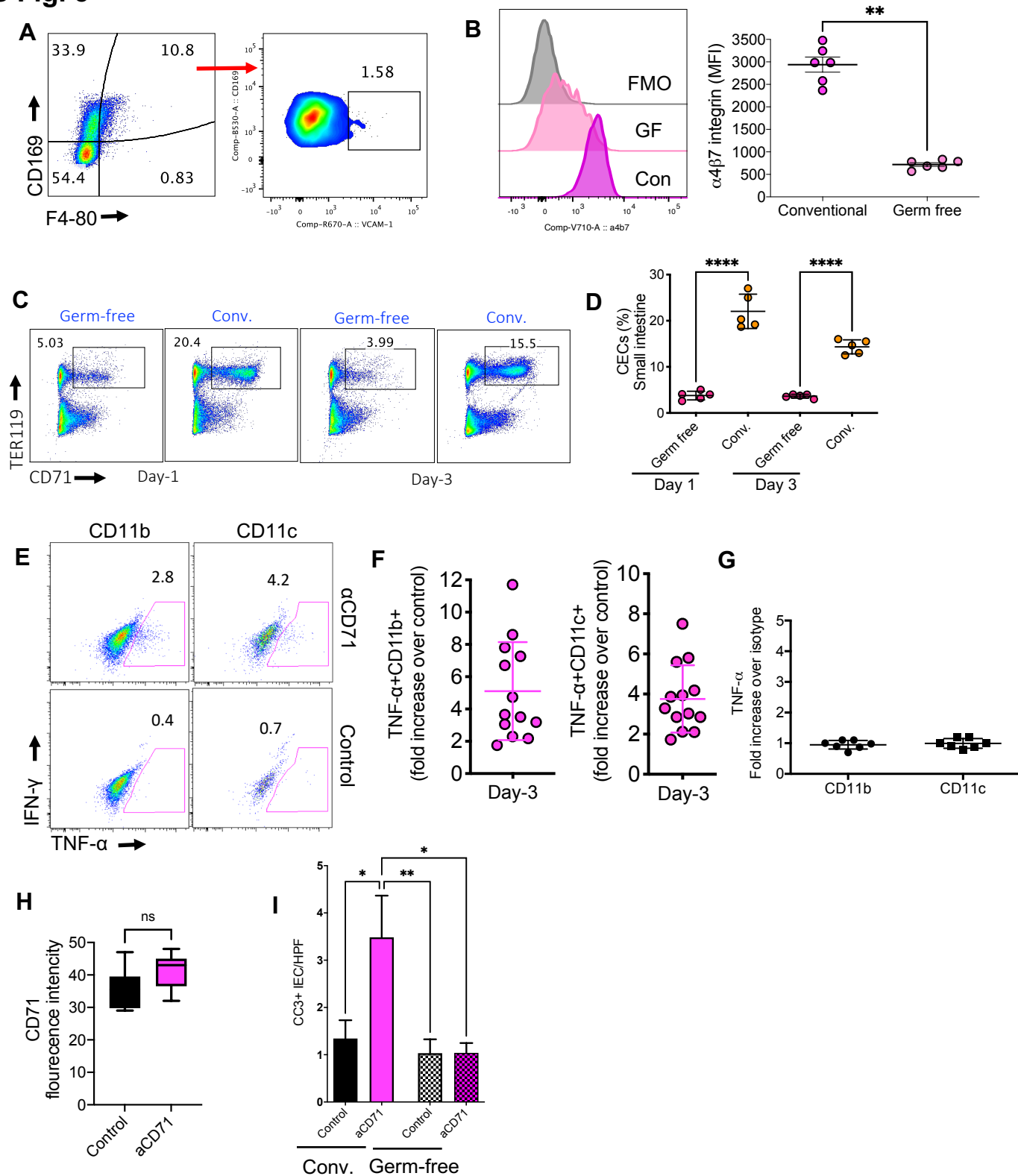

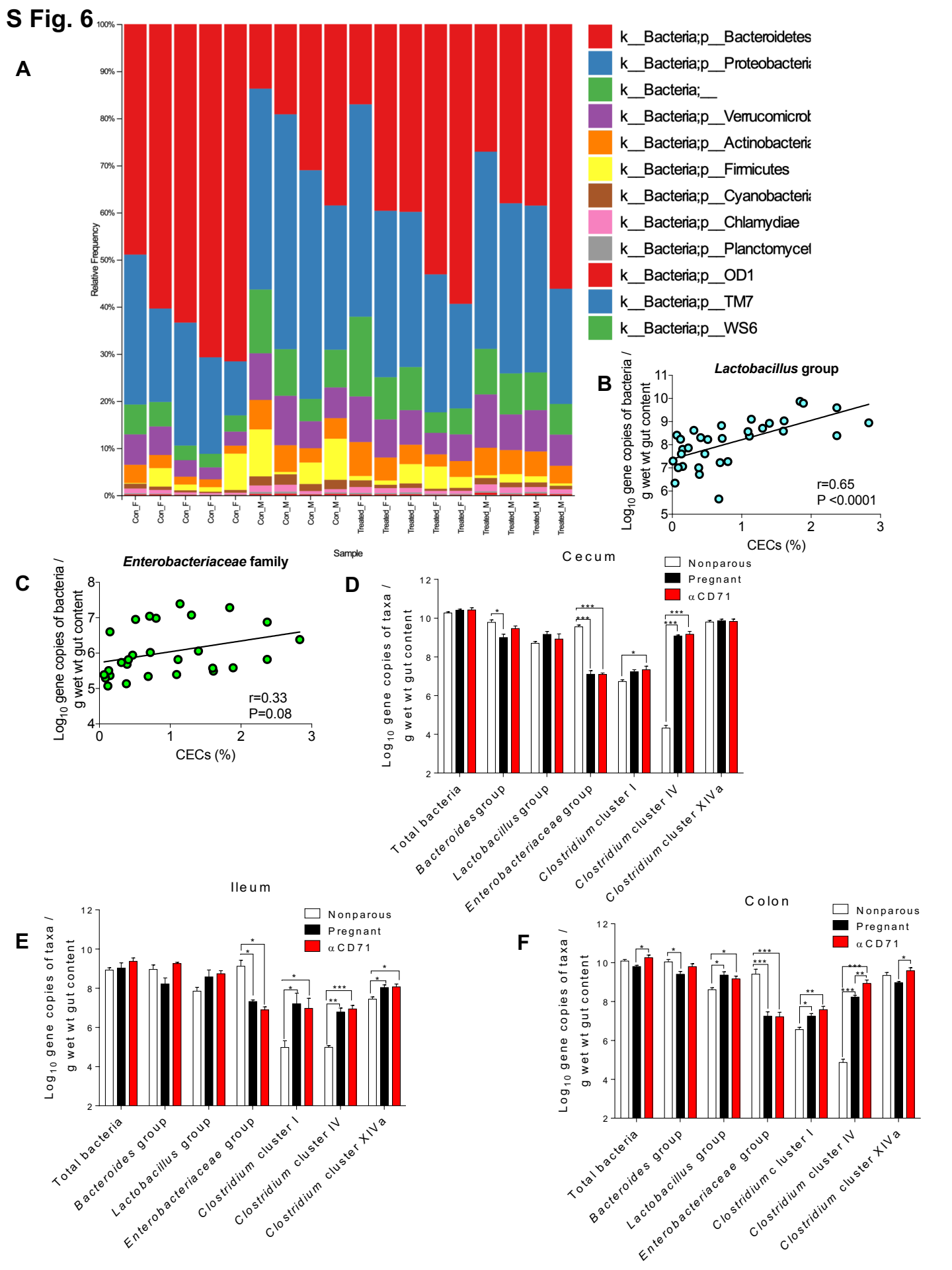

S Fig. 7

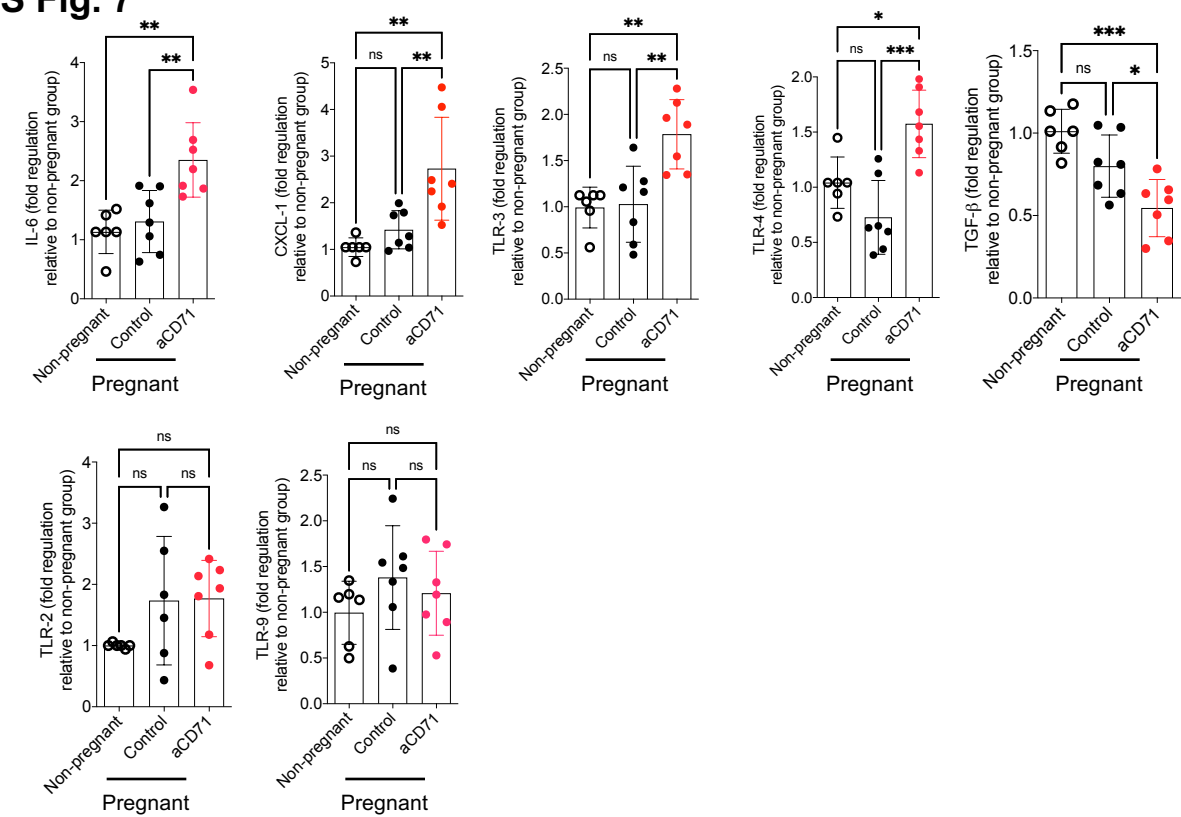

**S Fig. 8**

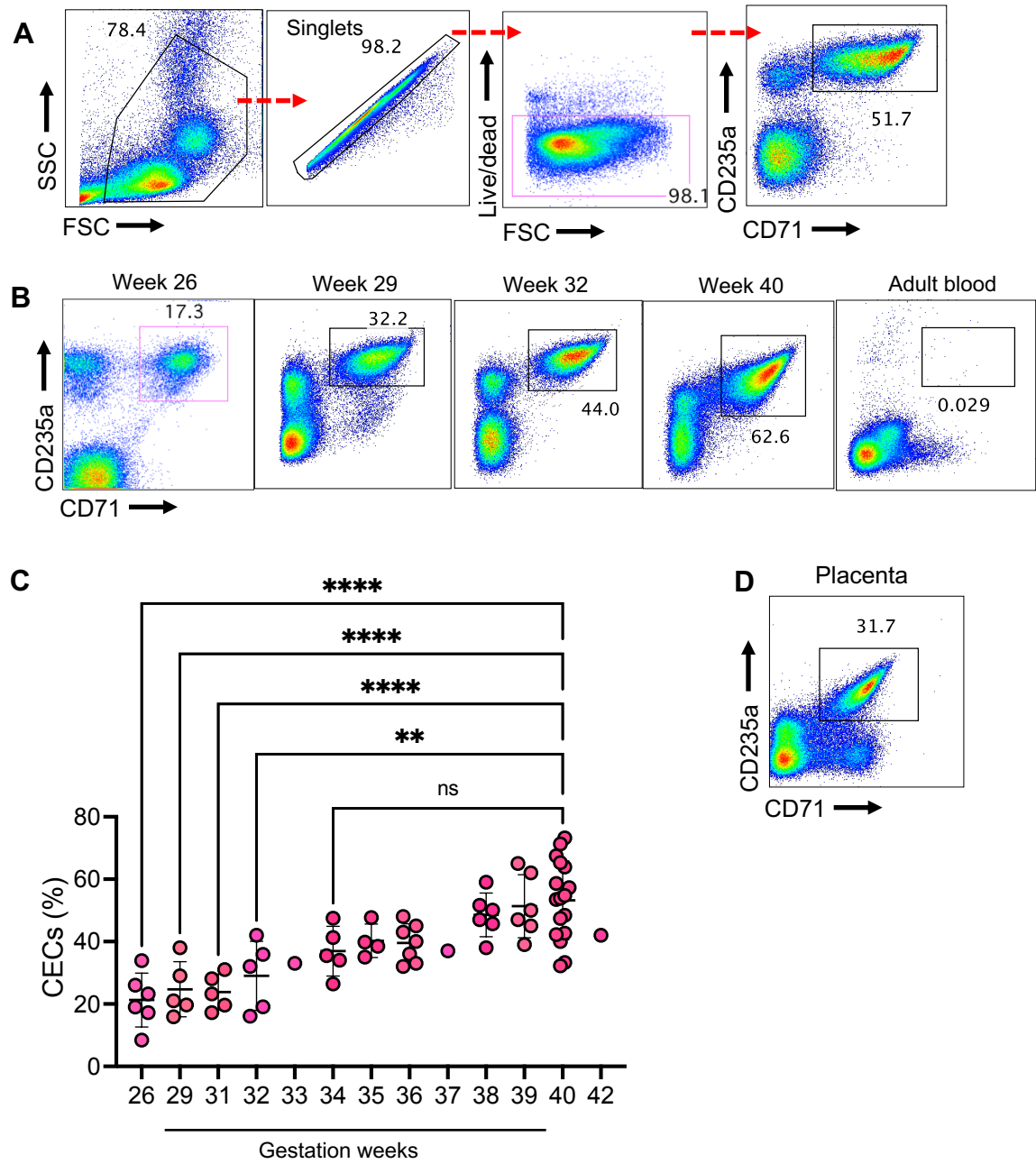

Supplement: Supplementary file 2 — Supplementary Material 1: Supplementary Figures S Fig 1. A) The gating strategy for the identification of CECs in intestinal tissues. B) Representative flow plots and cumulative data of the % of CECs in the spleen, small intestines, and colon tissues of 4-day-old control mice vs those treated with the anti-CD71 antibody a day earlier. C) Gene expression assay for detection of α4β7 integrin, and D) its ligand MAdCAM-1 in small intestinal tissues of control or CECs depleted animals. E) Correlation analysis of % CECs with the intensity of α4β7 in splenic CECs in 3-day-old mice. F) Representative flow cytometry plots showing the gating strategy for central macrophages (CD11b-CD169+F4-80+) in the small intestine of a 3-day-old mouse. G) Representative flow plots of central macrophages in a 3-day-old germ-free mouse. Results are presented as SD and P values were calculated using two tailed, Mann–Whitney t test (B-D) or the Spearmen correlation analysis (E). P < 0.05 (*). Anti-CD71 (aCD71). not significant (ns). S Fig. 2. Representative immunofluorescence staining (IF) plots at different indicated magnifications illustrating the presence of erythropoiesis niches in submucosal tissues of small intestine. DAPI (blue), TER119 (red), F4/80 (green). S Fig. 3. Representative IF plots at different indicated magnifications illustrating the presence of erythropoiesis niches in the villus of small intestine. DAPI (blue), TER119 (red), F4/80 (green). S Fig. 4. A) Representative IF plots illustrating the presence of mature red blood cells, without nuclei, in venules of small intestine. B) Representative IF plot illustrating the presence of erythropoiesis niches in the spleen of a neonatal mouse. DAPI (blue), TER119 (red), F4/80 (green). S Fig. 5. A) Representative flow cytometry plot of central macrophages in a germ-free mouse. B) Representative histogram and cumulative data of α4β7 expression in intestinal CECs of 3-day old conventional and germ-free mice. C) Representative flow cyto [file 40168_2024_1859_MOESM1_ESM.pdf]
