# Supplementary material for: CD71 + erythroid cells promote intestinal symbiotic microbial communities in pregnancy and neonatal period
Source: Microbiome. 2024 Jul 30;12:142. doi: 10.1186/s40168-024-01859-0 (PMC11290123; doi:10.1186/s40168-024-01859-0)
Supplement: Supplementary file 3 — Supplementary Material 2: S Table 1 [file 40168_2024_1859_MOESM2_ESM.pdf]

S. Table 1. TaqMan primer assays used in the gene expression experiment.

Gene symbol	Gene name	Assay number
Tlr2	Toll-like receptor 2	Mm01213946_g1
Tlr4	Toll-like receptor 4	Mm00445273_m1
Tlr5	Toll-like receptor 5	Mm00546288_s1
Tlr9	Toll-like receptor 9	Mm00446193_m1
Camp	Cathelicidin antimicrobial peptide	Mm00438285_m1
Mbd1	Mouse β-Defensin 1	Mm00522100_m1
Defa1	Defensin, alpha 1	Mm02524428_g1
Defa5	Defensin, alpha, 5	Mm00651548_g1
Mmp7	Matrix metallopeptidase 7	Mm00487724_m1
Ocln	Occludin	Mm00500912_m1
Tjp1	Tight junction protein 1	Mm00493699_m1
Tgfb1	Transforming growth factor, beta 1	Mm01178820_m1
Smad2	SMAD family member 2	Mm00487530_m1
Smad3	SMAD family member 3	Mm01170760_m1
Ccl2	Chemokine (C-C motif) ligand 2	Mm99999056_m1
Ccr7	Chemokine (C-C motif) receptor 7	Mm00432608_m1
Cxcl1	Chemokine (C-X-C motif) ligand 1	Mm04207460_m1
Cxcl2	Chemokine (C-X-C motif) ligand 2	Mm00436450_m1
Il6	Interleukin 6	Mm00446190_m1
Maea	Macrophage erythroblast attacher	Mm00491367_m1
Cdh1	E-cadherin	Mm01247357_m1
