# Supplementary material for: CD71 + erythroid cells promote intestinal symbiotic microbial communities in pregnancy and neonatal period
Source: Microbiome. 2024 Jul 30;12:142. doi: 10.1186/s40168-024-01859-0 (PMC11290123; doi:10.1186/s40168-024-01859-0)
Supplement: Supplementary file 4 — Supplementary Material 3: S Table 2 [file 40168_2024_1859_MOESM3_ESM.pdf]

S Table 2. Median relative abundance of dominant bacterial taxa at phylum, order and family levels in colon contents collected from nonporous, pregnant and pregnant anti-CD71 treated female mice.

**Bacterial taxa**	**Nonparous**	**Pregnant Control**	**Anti-CD71 treated**
**Actinobacteria**	**0.12 (0.00 - 0.55)^{A}**	**14.36 (0.77 - 27.92)^{B}**	**1.40 (0.91 - 3.64)^{A}**
Bifidobacteriales	0.06 (0.00 - 0.32)^{A}	13.05 (0.05 - 24.67)^{B}	0.00 (0.00 - 0.27)^{A}
Bifidobacteriaceae	0.06 (0.00 - 0.32)^{A}	13.05 (0.05 - 24.67)^{B}	0.00 (0.00 - 0.27)^{A}
Coriobacteriales	0.06 (0.00 - 0.23)^{A}	1.31 (0.72 - 3.26)^{B}	1.07 (0.62 - 1.70)^{B}
Coriobacteriaceae	0.06 (0.00 - 0.23)^{A}	1.31 (0.72 - 3.26)^{B}	1.07 (0.62 - 1.70)^{B}
**Bacteroidetes**	**61.65 (51.94 - 68.06)^{A}**	**36.64 (29.19 - 43.49)^{B}**	**22.02 (20.97 - 33.28)^{B}**
Bacteroidales	61.65 (51.94 - 68.06)^{A}	36.64 (29.19 - 43.49)^{B}	22.02 (20.97 - 33.28)^{B}
Bacteroidaceae	44.72 (26.64 - 50.07)^{A}	6.60 (2.11 - 8.02)^{B}	3.95 (1.57 - 5.98)^{B}
Porphyromonadaceae	8.77 (8.01 - 9.97)^{A}	0.50 (0.42 - 1.30)^{B}	0.75 (0.36 - 1.14)^{B}
S24_7	13.37 (3.54 - 16.45)^{A}	27.19 (26.49 - 36.47)^{B}	20.09 (15.51 - 28.67)^{AB}
**Firmicutes**	**30.74 (27.59 - 37.82)^{A}**	**42.14 (41.21 - 60.61)^{B}**	**70.83 (63.02 - 76.62)^{C}**
Lactobacillales	2.36 (1.46 - 4.99)^{A}	31.20 (24.06 - 34.42)^{B}	9.03 (4.63 - 21.91)^{A}
Lactobacillaceae	2.28 (1.39 - 4.93)^{A}	31.18 (24.02 - 34.40)^{B}	9.02 (4.63 - 21.91)^{A}
Clostridiales	26.13 (25.75 - 35.46)^{A}	18.07 (9.95 - 26.16)^{A}	54.97 (51.91 - 64.02)^{B}
un_Clostridiales	0.00 (0.00 - 4.91)^{A}	3.58 (2.94 - 7.15)^{B}	20.39 (13.51 - 21.01)^{C}

un_Clostridiales	0.12 (0.00 - 0.18)^{A}	6.33 (3.05 - 7.61)^{B}	16.00 (13.93 - 19.99)^{C}
Lachnospiraceae	25.95 (25.75 - 35.34)^{A}	5.87 (2.32 - 6.57)^{B}	11.17 (10.05 - 16.58)^{C}
Ruminococcaceae	0.00 (0.00 - 4.18)^{A}	1.78 (1.36 - 3.56)^{B}	6.47 (6.25 - 10.08)^{C}
**Proteobacteria**	**5.48 (3.99 - 6.99)^{A}**	**0.14 (0.09 - 0.49)^{B}**	**0.03 (0.02 - 0.31)^{B}**
Enterobacteriales	0.83 (0.69 - 0.96)^{A}	0.02 (0.01 - 0.09)^{B}	0.02 (0.01 - 0.022)^{B}
Enterobacteriaceae	0.83 (0.69 - 0.96)^{A}	0.02 (0.01 - 0.09)^{B}	0.02 (0.01 - 0.022)^{B}
Burkholderiales	1.89 (1.86 - 3.03)^{A}	0.12 (0.007 - 0.48)^{B}	0.00 (0.00 - 0.17)^{B}
Alcaligenaceae	1.89 (1.86 - 3.03)^{A}	0.12 (0.007 - 0.48)^{B}	0.00 (0.00 - 0.17)^{B}
**Verrucomicrobia**	**1.82 (0.00 - 3.06)^{A}**	**0.00 (0.00 – 0.00)^{A}**	**0.00 (0.00 - 0.007)^{A}**
Verrucomicrobiales	1.82 (0.00 - 3.06)^{A}	0.00 (0.00 – 0.00)^{A}	0.00 (0.00 - 0.007)^{A}
Verrucomicrobiaceae	0.91 (0.00 - 2.44)^{A}	0.00 (0.00 – 0.00)^{A}	0.00 (0.00 - 0.003)^{A}
**Deferribacteres**	**0.19 (0.00 - 0.31)^{A}**	**0.02 (0.003 - 0.03)^{B}**	**0.12 (0.06 - 0.18)^{A}**
Deferribacterales	0.19 (0.00 - 0.31)^{A}	0.02 (0.003 - 0.03)^{B}	0.12 (0.06 - 0.18)^{A}
Deferribacteraceae	0.19 (0.00 - 0.31)^{A}	0.02 (0.003 - 0.03)^{B}	0.12 (0.06 - 0.18)^{A}

Results are presented as medians(interquartile range). Comparisons were performed by Kruskal-Wallis test followed by Wilcoxon rank sum test. Experimental groups within the same bacterial taxa with unlike letters were significantly different ($P<0.05$).
